# Studies toward bivalent κ opioids derived from salvinorin A: heteromethylation of the furan ring reduces affinity

**DOI:** 10.3762/bjoc.9.328

**Published:** 2013-12-20

**Authors:** Thomas A Munro, Wei Xu, Douglas M Ho, Lee-Yuan Liu-Chen, Bruce M Cohen

**Affiliations:** 1 McLean Hospital, Belmont, MA 02478, USA and Department of Psychiatry, Harvard Medical School, Boston, MA 02215, USA; 2 School of Chemistry and Bio21 Institute, University of Melbourne, Parkville 3010, Australia; 3 Center for Substance Abuse Research and Department of Pharmacology, Temple University School of Medicine, Philadelphia, PA 19140, USA; 4 Department of Chemistry and Chemical Biology, Harvard University, Cambridge MA 02138, USA

**Keywords:** allotopic, bivalent ligand, designed multiple ligand, JDTic, κ-opioid receptor, natural products, Salvinorin A

## Abstract

The recent crystal structure of the κ-opioid receptor (κ-OR) revealed, unexpectedly, that the antagonist JDTic is a bivalent ligand: in addition to the orthosteric pocket occupied by morphinans, JDTic also occupies a distinct (allotopic) pocket. Mutagenesis data suggest that salvinorin A (**1**) also binds to this allotopic pocket, adjacent to the aspartate residue that anchors the basic nitrogen atom of classical opiates (Asp138). It has been suggested that an H-bond donor appended to **1** might interact with Asp138, increasing affinity. Such a bivalent ligand might also possess altered functional selectivity. Based on modeling and known *N*-furanylmethyl opioid antagonists, we appended H-bond donors to the furan ring of **1**. (Dimethylamino)methyl groups at C-15 or C-16 abolished affinity for κ-OR. Hydroxymethylation at C-16 was tolerated, but 15,16-bis-hydroxymethylation was not. Since allosteric modulators may go undetected in binding assays, we also tested these and other low-affinity derivatives of **1** for allosteric modulation of dynorphin A in the [^35^S]GTPγS assay. No modulation was detected. As an alternative attachment point for bivalent derivatives, we prepared the 2-(hydroxyethoxy)methyl ether, which retained high affinity for κ-OR. We discuss alternative design strategies for linked, fused or merged bivalent derivatives of **1**.

## Introduction

**Figure 1:**
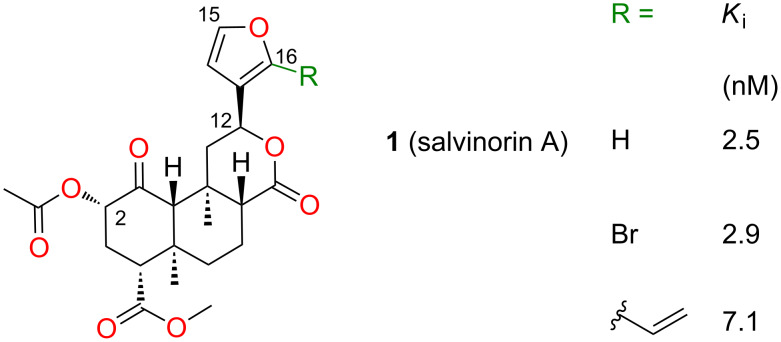
Binding affinities of salvinorin A (**1**) and furan derivatives for κ-OR [5].

The structure–activity relationships of salvinorin A (**1**), a potent and selective κ (kappa) opioid, have been extensively investigated [1]. Modifications at C-2 have yielded compounds with increased potency and duration of action [2–3], and other compounds with unprecedented functional selectivity at the μ (mu) opioid receptor (μ-OR) [4]. However, modifications at other positions substantially reduce or eliminate affinity for opioid receptors [1]. In particular, most modifications of the furan ring tested to date dramatically reduce affinity for κ-OR [1], although small substituents at C-16 have little effect (Figure 1). Unexpectedly, epimerization at C-12, inverting the configuration of the furan ring, reduces efficacy in G-protein activation [5], but not in arrestin recruitment, resulting in functional selectivity (biased agonism) [6].

**Figure 2:**
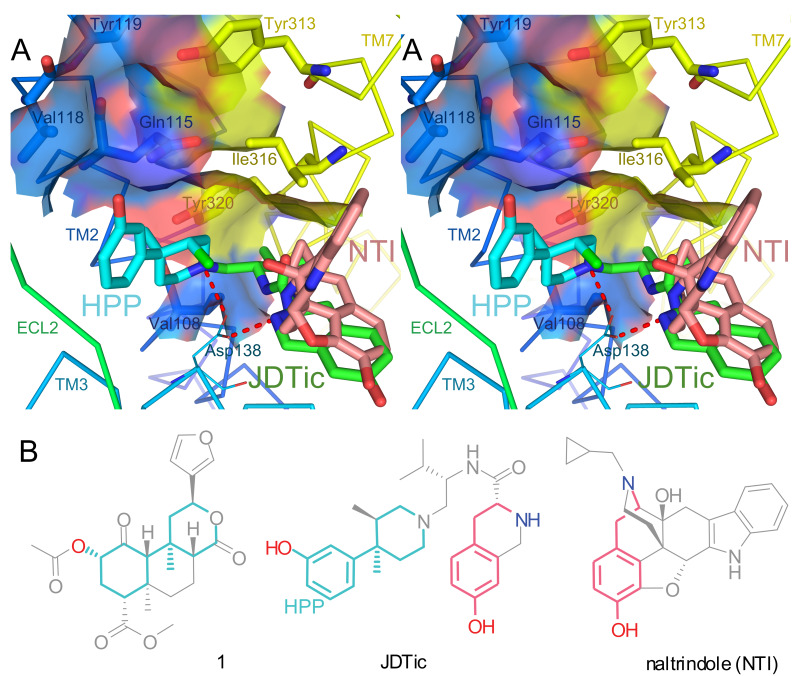
Crystal structure of κ-OR in complex with JDTic compared to naltrindole’s binding pose in δ-OR. A: Cross-eyed stereoview of the crystal structure of κ-OR (PDB 4DJH). All residues known to be required for high-affinity binding of **1** (mutation reproducibly reduces affinity ≥10-fold [7–9]) are shown with thick bonds and water-accessible surfaces. JDTic is shown in green with the hydroxyphenylpiperidine substructure (HPP) in light blue. Ionic H-bonds from JDTic to Asp138^3.32^ are shown in red. The binding pose of naltrindole (NTI, pink) to δ-OR is also superimposed (PDB 4EJ4). B: Structural similarities: atoms in **1** common to the HPP substructure of JDTic are shown in light blue; superimposable portions of naltrindole and JDTic are shown in pink.

Extensive receptor mutagenesis data are available on the interactions of κ-OR with **1** and derivatives [1,7–9]. In Figure 2A, the crystal structure of κ-OR in complex with the selective antagonist JDTic is shown. Essential residues for high affinity binding of **1**, where mutation reproducibly reduces affinity ≥10-fold, are shown with thick bonds for emphasis [7–9]. These key residues are all located in transmembrane helices (TMs) 2 and 7. The surfaces of these residues are contiguous, forming one face of a deep pocket between TMs 2, 3, and 7. Mutations of many other residues outside this pocket (TMs 1, 3, 5, 6, 7, and extracellular loop 2) failed to substantially reduce affinity for **1** [1,7–9]. Collectively, these results provide compelling evidence that **1** binds to this face of the binding pocket. The plausibility of this proposal is strengthened by the position of the hydroxyphenylpiperidine moiety of JDTic in the crystal structure (HPP, light blue). The HPP moiety and its *N*-substituent interact with five of the seven key residues for binding of **1**: Val108^2.53^, Gln115^2.60^, Val118^2.63^, Ile316^7.39^, and Tyr320^7.43^ (superscripts refer to Ballesteros–Weinstein numbering) [7]. Intriguingly, **1** contains a substructure very similar to HPP (light blue in Figure 2B). Given that HPP is a near substructure of **1** and binds to these same key residues, it is tempting to speculate that the binding pose of **1** may be similar.

By contrast, the morphinan naltrindole (shown in pink) binds to a different pocket of δ-OR among TMs 3, 6, and 7 [10]; β-funaltrexamine adopts a near-identical pose in complex with μ-OR (not shown) [11]. Interestingly, JDTic occupies this morphinan pocket as well. The tetrahydroisoquinoline moiety of JDTic adopts a pose almost identical to that of naltrindole; the superimposable atoms are shown in pink in Figure 2B [10]. JDTic is thus a bivalent ligand, containing two linked pharmacophores [12], also known as a linked multiple ligand [13]. Bivalent ligands can exhibit greatly increased potency and unusual functional selectivity [12]. Consistent with this, JDTic is extremely potent (*K*_e_ < 40 pM) [14]; also, despite acting as an antagonist toward other κ-OR-mediated signaling pathways [7], JDTic reportedly activates JNK1 (MAPK8), causing extremely prolonged desensitization of κ-OR [15].

These two binding pockets are almost entirely separate, but overlap around Tyr320^7.43^. This residue interacts with JDTic [7], and the corresponding residue (Tyr^7.43^) also interacts with naltrindole in δ-OR [10] and β-funaltrexamine in μ-OR [11].

Interestingly, Tyr320^7.43^ and two adjacent residues (Val108^2.53^ and Ile316^7.39^) are also key residues for the binding of **1** [7]. Together, they surround the bottom of the binding pocket, strongly suggesting that an extremity of **1** occupies this space, overlapping with both the morphinan and hydroxyphenylpiperidine binding sites.

Immediately adjacent to these residues is Asp138^3.32^, a critical residue for binding of morphinans and related opioids. Asp^3.32^ forms strong ionic H-bonds (‘salt bridges’) to the basic nitrogen atoms found in almost all opioids, as in the crystal structures of JDTic (red in Figure 2A), naltrindole [10], β-funaltrexamine [11], and the NOP antagonist C-24 [16]. Beyond opioids, ionic H-bonds to Asp^3.32^ are conserved across biogenic amine receptors; indeed, this residue^3.32^ interacts with the ligand in almost all GPCR crystal structures reported to date [17].

In summary, **1** appears to bind adjacent to Asp138^3.32^, but not to interact with it strongly: mutation of this residue either has little effect or actually increases affinity [7–9]. Based on this proximity, Kane proposed appending a positively-charged moiety to **1**, thereby creating an ionic H bond to Asp138^3.32^ that should increase affinity [18]. Like JDTic, this ligand would be bivalent. Kane also proposed linking to fragments of other κ-opioids such as arylacetamides, creating additional interactions. By this strategy, “designed multiple ligands” of several kinds could be obtained: linked, fused or merged [13].

**Figure 3:**
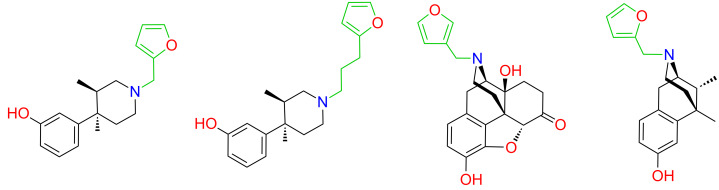
Previously reported *N*-furanylalkyl opioid antagonists [19–20].

A suitable attachment point and linker for this second moiety would also be required. Docking **1** to an early rhodopsin-based homology model of κ-OR, Kane proposed a binding pose qualitatively similar to that of the HPP moiety of JDTic, placing the furan ring near Tyr320^7.43^ [8,18]. Indeed, at the time we commenced our work, all published mutagenesis-based models placed the furan ring in contact with Tyr320^7.43^ [1]. This interaction is plausible in light of the structure–activity relationships of opioid antagonists. In JDTic, the *N*-substituent of the HPP moiety interacts with Tyr320^7.43^ [7], and *N*-furanylalkyl substitution of this scaffold yields potent opioid antagonists (Figure 3) [19]. Likewise, the *N*-substituents of naltrindole and β-funaltrexamine also interact with Tyr^7.43^ [10–11], and *N*-furanylmethyl substitution of morphinans and related scaffolds consistently yields potent opioid antagonists [20]. These convergent results firmly establish that residues adjacent to Asp^3.32^, including Tyr^7.43^, interact favorably with furan substituents; thus, models placing the furan ring of **1** in this region are plausible. The region appears to be quite tolerant to the position, orientation and substitution of the furan ring [20].

We therefore selected the furan ring as an attachment point for initial explorations of Kane’s bivalent ligand concept. Given the tolerance of **1** to modification at C-16 noted above, we chose to append H-bond donors (amino and hydroxy groups) at this position. Based on previous high-affinity ligands with a furanylmethyl substituent (Figure 3), we decided to use a short linker.

As an alternative approach, bivalent ligands with much longer linkers can bind simultaneously to both protomers in a receptor dimer, allowing selective targeting of specific receptor oligomers [21]. We also sought to provide a suitable attachment point for such linkers based on our previous studies of C-2 alkoxymethyl ethers [22–23], which have higher tolerance to alkyl chain extension [24] than other derivatives of **1** [1].

## Results

### Synthesis

**Scheme 1:**
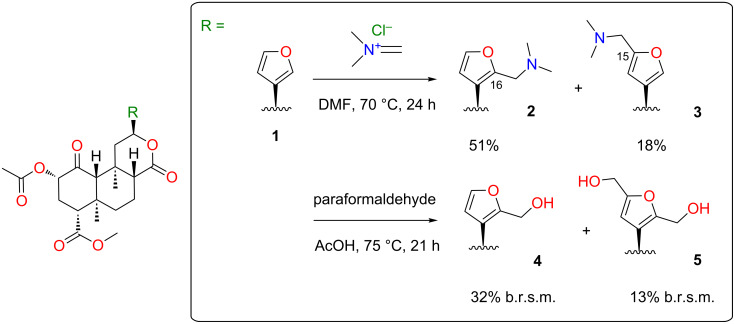
Syntheses of heteromethylated derivatives of **1**. b.r.s.m. = based on recovered starting material.

Initial attempts to form the 16-dimethylaminomethyl derivative **2** by treatment of **1** with dimethylamine and paraformaldehyde in acetic acid [25] gave no desired product. However, *N*,*N*-dimethylmethyleneiminium chloride [26] in dimethylformamide gave **2** and the 15-regioisomer **3** in good total yield (Scheme 1). Treatment of **2** with HCl in Et_2_O gave the hydrochloride salt, but the same treatment of **3** gave a cloudy suspension, with decomposition products evident in the ^1^H NMR spectrum.

We next targeted hydroxymethyl substituents. Treatment of **1** with aqueous formaldehyde (40% w/v) and Amberlyst-15^®^ resin at room temperature [27] gave complex mixtures of products. However, heating **1** with paraformaldehyde in acetic acid [28] gave **4** and **5**, albeit in low yields based on recovered starting material (b.r.s.m.) (Scheme 1). Prolonging the reaction or raising temperatures did not increase yields. No 15-hydroxymethyl derivative was isolated. Excess paraformaldehyde tended to precipitate during chromatography, blocking the column. This was therefore extracted from the crude product with hot water. Our results conflict with a previous report that heating **1** in acetic acid led to deacetylation and epimerization [29]. In our hands, **1** was freely soluble in glacial acetic acid and was recovered unchanged after heating overnight at reflux.

**Scheme 2:**
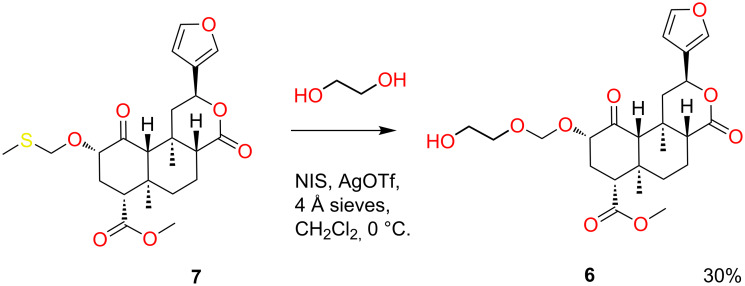
Synthesis of (2-hydroxyethoxy)methyl ether **6**.

As an alternative linker attachment point, (2-hydroxyethoxy)methyl ether **6** was prepared from the known methylthiomethyl ether **7** using a slight modification of previous conditions (Scheme 2) [24].

### Structure elucidation

**Figure 4:**
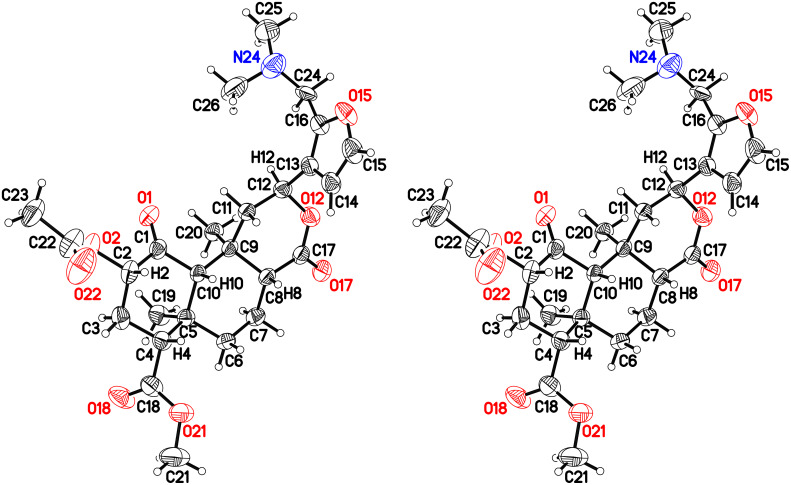
Crystal structure of **2** with 50% probability thermal displacement ellipsoids (cross-eyed stereoview). For clarity, only one of the two observed orientations for the disordered dimethylamino group is shown. For the other orientation and partially-occupied ethanol solvate, see Supporting Information File 1, Figure S1. For coordinates, see Supporting Information File 2 or CCDC 970639.

The molecular formula of **2** and **3** was established by HRMS, confirming the addition of a dimethylaminomethyl group in each case. The structure of **2** was established by X-ray crystallography (Figure 4) after recrystallization from ethanol/hexanes.

**Figure 5:**
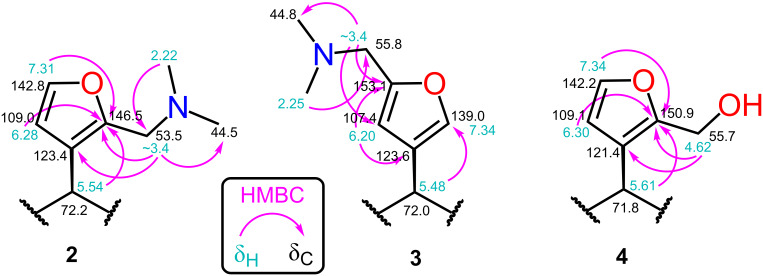
Key ^1^H–^13^C HMBC correlations.

Consistent with the crystal structure, comparison of the ^1^H NMR spectrum of **2** with the fully assigned spectrum of **1** [30–31] revealed new dimethylamino (δ 2.22, s, 6H) and aminomethylene (δ ~3.4, m, 2H) signals and the loss of one furan oxymethine. The remaining oxymethine signal (δ 7.31) was a doublet with a typical vicinal coupling constant (1.7 Hz) to H-14 (δ 6.28), confirming that they were adjacent. The multiple-bond ^1^H–^13^C (HMBC) correlations for **2** shown in Figure 5 confirm substitution at C-16.

The structure of the other regioisomer, **3**, was confirmed using NMR data. The oxymethine signal (δ 7.34) showed only long-range couplings (*J* = 0.8 Hz), confirming that it was isolated. The HMBC correlations (Figure 5) confirmed substitution at C-15.

The molecular formula of **4**, established by HRMS, confirmed the addition of a hydroxymethyl group. Like the C-16-substituted **4**, the two furan peaks in the ^1^H NMR spectrum were doublets with a typical vicinal coupling constant (1.9 Hz). The same HMBC correlations were observed as for **2** (Figure 5), confirming substitution at C-16. The molecular formula of **5**, established by HRMS, indicated the addition of two hydroxymethyl groups. The remaining furan oxymethine signal seen in the ^1^H NMR spectrum of **4** was lost, leaving an H-14 singlet (δ 6.21), confirming substitution at C-15 and C-16. An oxymethylene triplet (δ 4.61, t, 2H) and doublet (δ 4.55, d, 2H) collapsed to a doublet and singlet, respectively, after washing with D_2_O.

The molecular formula of (2-hydroxyethoxy)methyl ether **6** was confirmed by HRMS. The structure was confirmed by comparison of ^1^H and ^13^C NMR spectra with the previously reported (2-methoxyethoxy)methyl ether [24].

### Bioassays

We next determined the binding affinities of these compounds in blinded tests for the human κ-OR expressed in Chinese hamster ovary cells (CHO-κ-OR). In an initial screening of the furan derivatives at 3 µM, only **4** caused ≥50% inhibition of [^3^H]diprenorphine binding. The *K*_i_ of **4** was 110 nM (Table 1), a substantial reduction in affinity relative to **1**. None of these compounds showed detectable affinity for µ- or δ-OR (*K*_i_ > 1 μM in all cases). Thus, **4** showed modest affinity and selectivity for κ-OR, while **2**, **3** and **5** showed no affinity for any opioid receptor. Follow-up testing in the [^35^S]GTPγS functional assay demonstrated that **4** was an agonist less potent than **1** at κ-OR, but giving an equal maximal response (Table 1). (2-Hydroxyethoxy)methyl ether **6** showed high affinity for κ-OR, but not for µ- or δ-OR (*K*_i_ > 1 μM), similar to other alkoxymethyl ethers [23–24].

**Table 1:** Binding affinity, potency and maximal response at κ-OR.

			*K_i_* ± SEM^a^	EC_50_ ± SEM^b^	*E_max_*^c^
	**R**		nM	nM	%

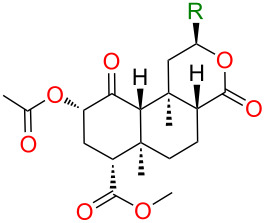	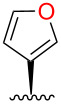	**1**	3.0^d^	7.6 ± 0.3	106
	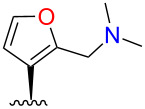	**2**	>1,000		
	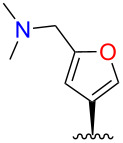	**3**	>1,000		
	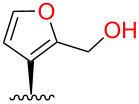	**4**	110 ± 10	345 ± 47	103
	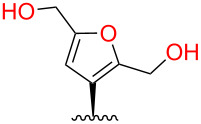	**5**	>1,000		
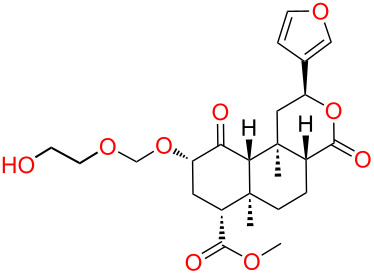		**6**	12.7 ± 1.3		
	U50,488H		1.2 ± 0.2	6.8 ± 1.1	100

^a^Displacement of [^3^H]diprenorphine from CHO cell membranes expressing human κ-OR. Results represent mean ± standard error of the mean for three independent experiments (*n* = 3) performed in duplicate. ^b^Potency in enhancement of [^35^S]GTPγS binding to CHO-κ-OR membranes (*n* = 3–5). ^c^Maximal [^35^S]GTPγS binding relative to U50,488H. ^d^As reported previously [6].

Despite the low affinities and potencies of the furan derivatives, we undertook further investigations. As discussed in the Introduction, the key residues for the binding of **1** are almost entirely separate from the morphinan binding pocket. Although it seems likely that the two pockets overlap, it is possible that they are separate, or allotopic [32]. If so, **1** could bind simultaneously with morphinans and act as an allosteric modulator. Indeed, it has been reported that **1** is a low-affinity allosteric modulator of µ-OR [33]. Since it is possible for allosteric ligands to bind, and to modulate signaling, without displacing orthosteric ligands such as diprenorphine [34], binding assays can give false negative results.

**Figure 6:**
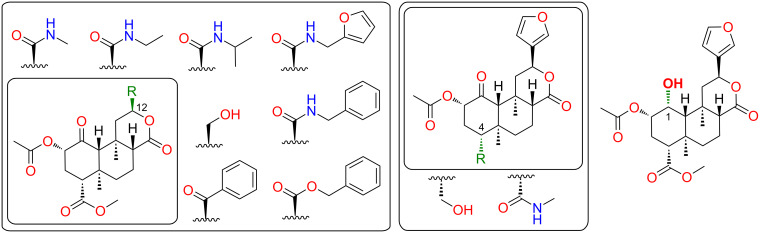
Known low-affinity derivatives of **1** screened in addition to **2**–**5** for negative allosteric modulation of dynorphin A at κ-OR in the [^35^S]GTPγS assay. No compound caused detectable antagonism at 3 μM.

We therefore tested whether the furan derivatives of **1** that failed to displace [^3^H]diprenorphine from κ-OR nonetheless modulated signaling. Given our interest in the development of κ-opioid antagonists [35], we tested for negative modulation. To maximize the physiological relevance of the assays, we used the endogenous agonist dynorphin A. In addition to **2**–**5**, we retested a number of derivatives of **1** we had previously found to exhibit negligible affinity for κ-OR; these featured modifications at C-12 [5], C-4 [36] and C-1 [31] (Figure 6).

In a primary screen at 3 μM, none of the compounds tested caused detectable antagonism of dynorphin A (10 nM) in the [^35^S]GTPγS assay (Supporting Information File 1, Table S1). By comparison, the positive control, JDTic, abolished response to dynorphin A. These results reveal no evidence of allosteric modulation, but do not exclude the possibility [34].

## Discussion

While the affinities of all these furan derivatives were low, they may nonetheless provide starting points for the development of bivalent derivatives. Installation of a leaving group on hydroxymethyl compound **4**, for instance, would permit coupling with diverse *N*-dealkylated opiate scaffolds. Alternative attachment points would also be worth exploring, such as the C-17 lactone, which tolerates reduction and alkylation [1]. As noted in the Introduction, there is compelling evidence that **1** binds adjacent to Asp138^3.32^, but the involvement of the furan ring is based on modeling rather than direct evidence, and is thus highly speculative. A subsequent model for the binding of **1** derivative RB-64 places the C-18 methyl ester rather than the furan ring close to Asp138^3.32^ [7]. In our previous studies, however, substitution of amine groups for the C-18 methyl ester abolished affinity for κ-OR [36]. Indeed, all modifications of this group tested to date greatly reduce affinity, in most cases abolishing binding altogether [1]. C-18 is thus not a promising attachment point. At the opposite extreme, the C-2 alkoxymethyl ether series is highly tolerant to alkyl chain extension. Compound **6** and the synthetic route used may prove useful for the attachment of very long linkers, as used with other scaffolds to bridge receptor dimers [21].

Rather than linking pharmacophores, another approach to bivalent ligands is to design hybrid structures, or merged multiple ligands [13]. As noted above, the HPP moiety of JDTic interacts with many of the key residues for binding of **1**, and shares a very similar substructure, suggesting that the binding pose of **1** may be similar. If so, converting the lactone to a cyclic amine may provide the desired H-bond to Asp138^3.32^ without the need for additional functional groups.

Our results provide no evidence of negative allosteric modulation of κ-OR by low-affinity derivatives of **1**, and the mutagenesis results discussed in the Introduction suggest some overlap with other opioids. However, given the report that **1** is an allosteric modulator of μ-OR [33], high-affinity μ-selective derivatives such as herkinorin [1] also represent potential scaffolds for bivalent and possibly bitopic (orthosteric–allosteric) derivatives [12].

## Conclusion

In conclusion, compelling evidence suggests that **1** binds to an allotopic pocket of κ-OR, leaving the classical opiate binding site largely unoccupied. This suggests the possibility of creating bivalent derivatives by linking these scaffolds, with the potential for dramatic increases in potency and altered functional selectivity. A suitable attachment point and linker remain to be determined. Our preliminary modifications at C-2, C-15 and C-16 yielded no potent derivatives, but may prove useful as intermediates in further exploration of this concept.

## Author Contributions

T.A.M. designed and prepared the compounds, wrote the manuscript and prepared the figures. W.X. and L.Y.L.C. performed and analyzed the bioassays. D.M.H. obtained the crystal structure. B.M.C. supervised and coordinated the project.

## Competing Interests

B.M.C., L.Y.L.C., and T.A.M. hold US patents on the preparation and therapeutic use of salvinorin A derivatives (7,629,475 and 8,492,564).

## Supporting Information

File 1 Synthetic procedures and ^1^H and ^13^C NMR spectra of all new compounds; HMQC and HMBC spectra from Figure 5; supporting Figure S1 and Table S1.

File 2 ^1^H and ^13^C NMR spectra of all new compounds in jcamp-dx format.

File 3 X-ray crystal structure of **2**.

File 4 Structure factors for **2**.
