# Supplementary material for: Studies toward bivalent κ opioids derived from salvinorin A: heteromethylation of the furan ring reduces affinity
Source: Beilstein J Org Chem. 2013 Dec 20;9:2916–24. doi: 10.3762/bjoc.9.328 (PMC3896271; doi:10.3762/bjoc.9.328)
Supplement: File 1 — Synthetic procedures and 1H and 13C NMR spectra of all new compounds; HMQC and HMBC spectra from Figure 5; supporting Figure S1 and Table S1. [file Beilstein_J_Org_Chem-09-2916-s001.pdf]

Studies toward bivalent κ opioids derived from salvinorin A: heteromethylation of the furan ring reduces affinity.

Thomas A. Munro, Wei Xu, Douglas M. Ho, Lee-Yuan Liu-Chen and Bruce M. Cohen.

Supporting Information

Table of contents

	Page
Experimental methods	2
References	7
Table S1	8
Figure S1	9
NMR spectra:	
**2**	10
**3**	15
**4**	19
**5**	24
**6**	26

Bioassays

Bioassays have been described in detail [1] and summarized [2] previously. Briefly, binding affinities at μ, δ, and κ opioid receptors were determined by competitive inhibition of [^{3}H]diprenorphine binding to membranes prepared from Chinese hamster ovary (CHO) cells stably transfected with human κ, rat μ, or mouse δ opioid receptors. Compounds were initially screened at 3 μM; those compounds causing >50% displacement of [^{3}H]diprenorphine (equivalent under the assay conditions to K_i < 1 μM) were tested further for determination of binding affinity (K_i), potency (EC_{50}) and efficacy (E_{max}). Positive controls were U50,488H (κ), DAMGO (μ), SNC80 (δ), and etorphine (μ/δ), which all caused > 90% displacement of [^{3}H]diprenorphine at 3 μM. Potencies and efficacies were determined by [^{35}S]GTPγS binding to membranes of CHO-hκ-OR cells, as described in detail elsewhere [1]. Testing was blinded: neither identity nor molecular mass were known to the testers.

General Synthetic Methods

Commercial reagents and solvents were used without further purification unless otherwise noted. Salvinorin A (**1**) was isolated from dried *Salvia divinorum* leaves as described previously [3]. Reactions were monitored by thin-layer chromatography (TLC) on silica gel using vanillin/H_2SO_4 in EtOH or Hanessian's stain [ammonium molybdate $(NH_4)_6Mo_7O_{24}\cdot 4H_2O$), cerium sulfate ($Ce(SO_4)_2$), and H_2SO_4 in H_2O], developed with a heat gun. Products were purified using automated flash chromatography with gradient elution on 35 μm silica gel. Melting points were determined at 2 °C/min. ^{1}H and ^{13}C NMR chemical shifts are referenced to residual **$CDCl_3$** (7.26 and 77 ppm).

Synthesis

(Dimethylamino)methylation of 1: *N,N*-dimethylmethyleneiminium chloride (35.8 mg, 382.6 μmol) and **1** (54.2 mg, 125.3 μmol) were sealed under Ar. DMF (2 mL) was added, and the solution stirred at 70 °C for 24 h, when TLC showed no **1**. After cooling to rt, sat. $NaHCO_3/H_2O$ was added dropwise with stirring, giving a thick precipitate. The mixture was diluted with EtOAc, washed with sat. $NaHCO_3/H_2O$ (×3) followed by brine, then dried (K_2CO_3), filtered and evaporated under reduced pressure. Flash chromatography (35 μm silica gel × 4 g) in 5–10% CH_3OH/CH_2Cl_2 and 0–30% CH_3OH/EtOAc gave **2** (31.4 mg, 64.1 μmol, 51%) and **3** (11.3 mg, 23 μmol, 18%). Crystals of an ethanol solvate of **2** were grown by dissolving in minimal hot ethanol. The solution was diluted to approximately 10% in hexanes, warmed until clear, then left to evaporate in a fumehood. **TLC** (30% CH_3OH/EtOAc, Hanessian's stain) hR_f = 83 (**1**), 49 (**2**), 26 (**3**).

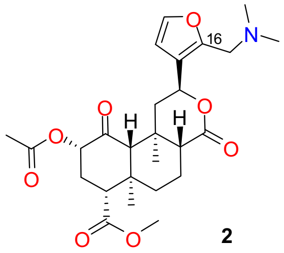

2

16-((dimethylamino)methyl)salvinorin A (2): colorless crystals, mp 156–158 °C (decomp.); **^{1}H NMR (300 MHz, $CDCl_3$)** δ 7.32–7.31 (m, 1H), 6.28 (d, *J* = 1.9, 1H), 5.55 (dd, *J* = 11.9, 5.1, 1H), 5.11 (t, *J* = 10.0, 1H), 3.71 (s, 3H), 3.42 (br s, 2H), 2.79–2.70 (m, 1H), 2.38–2.20 (m, 4H), 2.22 (s, 6H), 2.18–2.08 (m, 2H), 2.17 (br s, 1H), 2.14 (s, 3H), 1.80–1.73 (m, 1H), 1.69–1.53 (m, 4H), 1.44 (s, 3H), 1.10 (s, 3H); **^{13}C NMR (75 MHz, $CDCl_3$)** δ 202.1, 171.6, 171.3, 169.8, 146.5, 142.8, 123.4, 109.0, 75.0, 71.9, 63.6, 53.5, 53.4, 51.9, 51.1, 44.5, 43.2, 42.0, 38.0, 35.6, 35.4, 30.7, 20.6, 18.1, 16.4, 15.1; **HRMS(ESI):** $[M+H]^+$ *m/z* 490.2442 (calcd for $C_{26}H_{35}NO_8$, 490.2435).

To form the hydrochloride, fresh HCl in Et_2O (1M, 350 µL, 1.3 eq) was added to **2** (132 mg, 270 µmol) in CH_2Cl_2 (3 mL). No precipitate was observed; evaporation (rotavap) gave a clear resin, freely soluble in CH_2Cl_2 and $CDCl_3$. Trituration in Et_2O gave **2**•HCl as colorless crystals (143 mg).

2•HCl: colorless crystals, mp 240–243 °C (decomp.); **^{1}H NMR (500 MHz, $CDCl_3$)** δ 12.53 (br s, 1H), 7.44 (d, *J* = 1.7, 1H), 6.34 (d, *J* = 1.6, 1H), 5.62 (dd, *J* = 12.2, 4.9, 1H), 5.16 (dd, *J* = 11.7, 8.3, 1H), 4.61 (d, *J* = 14.3, 1H), 3.99 (d, *J* = 14.2, 1H), 3.70 (s, 3H), 3.03 (dd, *J* = 11.9, 1.9, 1H), 2.87 (dd, *J* = 12.1, 4.8, 1H), 2.82 (s, 6H), 2.49 (br s, 1H), 2.38 (dd, *J* = 13.2, 4.9, 1H), 2.32–2.23 (m, 2H), 2.19–2.14 (m, 2H), 2.15 (s, 3H), 1.75–1.70 (m, 2H), 1.66–1.55 (m, 3H), 1.45 (s, 3H), 1.10 (s, 3H).

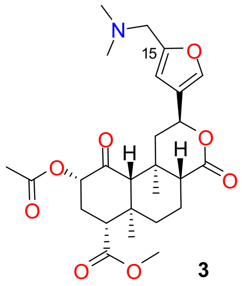

3

15-((dimethylamino)methyl)salvinorin A (3): amber resin; **^{1}H NMR (300 MHz, $CDCl_3$)** δ 7.34 (t, *J* = 0.8, 1H), 6.20 (d, *J* = 0.5, 1H), 5.48 (dd, *J* = 11.6, 5.1, 1H), 5.18–5.11 (m, 1H), 3.72 (s, 3H), 3.43 (s, 2H), 2.79–2.69 (m, 1H), 2.49 (dd, *J* = 13.4, 5.2, 1H), 2.33–2.28 (m, 2H), 2.25 (s, 6H), 2.19–2.13 (m, 1H), 2.17 (s, 3H), 2.15 (br s, 1H), 2.08–2.03 (m, 2H), 1.81–1.76 (m, 1H), 1.71–1.52 (m, 3H), 1.44 (s, 3H), 1.11 (s, 3H); **^{13}C NMR (75 MHz, $CDCl_3$)** δ 202.0, 171.5, 171.1, 169.9, 153.1, 139.0, 125.6, 107.4, 74.9, 72.0, 63.9, 55.5, 53.5, 51.9, 51.3, 44.8, 43.2, 42.0, 38.1, 35.4, 30.7, 20.5, 18.1, 16.3, 15.1; **HRMS(ESI):** $[M+H]^+$ *m/z* 490.2442 (calcd for $C_{26}H_{35}NO_8$, 490.2435).

Hydroxymethylation of 1: Glacial acetic acid (5 mL) was added to **1** (305 mg, 705 μmol) and paraformaldehyde (115 mg, 3.8 mmol, 5.4 eq) and stirred at 75 °C for 20 h; the initial suspension cleared within 3 h. Starting material was still present by TLC (EtOAc) at 20 h. The solution was evaporated under reduced pressure, water (10 mL) was added, and the suspension stirred at 95 °C for 1 h to remove excess formaldehyde. The supernatant was immediately decanted while hot. The solid was dried under reduced pressure to give a brown foam (283 mg). Flash chromatography (35 μm silica gel × 8 g, 20–100% EtOAc/hexanes gradient) gave recovered **1** (102 mg) along with **4** (70.1 mg, 151.6 μmol, 21% [32% b.r.s.m.]) and **5** (29.4 mg, 59.6 μmol, 8% [13% b.r.s.m.]). **TLC** (EtOAc) hR_f = 77 (**1**), 63 (**4**), 27 (**5**).

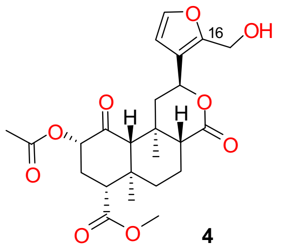

4

16-(hydroxymethyl)salvinorin A (4): colorless crystals, mp 215–217 °C (decomp.); **^{1}H NMR (300 MHz, $CDCl_3$) δ** 7.34 (d, *J* = 1.9, 1H), 6.30 (d, *J* = 1.9, 1H), 5.61 (dd, *J* = 11.8, 5.1, 1H), 5.16–5.09 (m, 1H), 4.69–4.57 (m, 2H), 3.73 (s, 3H), 2.74 (dd, *J* = 15.7, 7.5, 1H), 2.43 (dd, *J* = 13.5, 5.2, 1H), 2.33–2.26 (m, 2H), 2.19 (br s, 1H), 2.22–2.15 (m, 2H), 2.16 (s, 3H), 2.11 (dd, *J* = 11.0, 3.0, 1H), 1.95–1.91 (m, 1H), 1.82–1.78 (m, 1H), 1.68–1.58 (m, 7H), 1.46 (s, 3H), 1.12 (s, 3H); **^{13}C NMR (75 MHz, $CDCl_3$)** δ 202.0, 171.5, 171.2, 170.0, 150.9, 142.2, 121.3, 109.1, 75.0, 71.8, 63.7, 55.7, 53.4, 51.9, 51.3, 43.5, 42.0, 38.0, 35.5, 30.7, 20.5, 18.1, 16.4, 15.1; **HRMS(ESI):** $[M+NH_4]^+$ *m/z* 480.2238 (calcd for $C_{24}H_{30}O_9$, 480.2228).

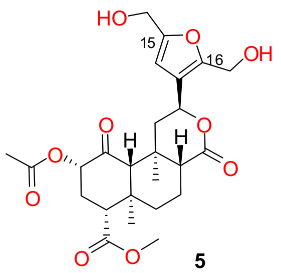

5

15,16-bis(hydroxymethyl)salvinorin A (5): amber resin; **^{1}H NMR (300 MHz, $CDCl_3$)** δ 6.21 (s, 1H), 5.57 (dd, *J* = 11.8, 5.2, 1H), 5.18–5.03 (m, 1H), 4.61 (t, *J* = 4.2, 2H), 4.55 (d, *J* = 5.1, 2H), 3.73 (s, 3H), 2.76 (dd, *J* = 9.1, 7.7, 1H), 2.41 (dd, *J* = 13.6, 5.2, 1H), 2.33–2.26 (m, 2H), 2.21–2.08 (m, 5H), 2.16 (s, 3H), 1.95–1.88 (m, 1H), 1.80 (dd, *J* = 9.9, 2.9, 1H), 1.71–1.54 (m, 6H), 1.45 (s, 3H), 1.11 (s, 3H); **^{13}C NMR (75 MHz, $CDCl_3$)** δ 202.0, 171.5, 171.1, 170.0, 153.8, 150.6, 122.2, 107.1, 75.1, 71.8, 63.9, 57.4, 55.9, 53.5, 52.0, 51.3, 43.5, 42.1, 38.1, 35.5, 30.7, 20.6, 18.1, 16.4, 15.2; **HRMS(ESI):** $[M+NH_4]^+$ *m/z* 510.2330 (calcd for $C_{25}H_{32}O_{10}$, 510.2334).

Salvinorin B (2-hydroxyethoxy)methyl ether (6): *N*-iodosuccinimide (NIS) was recrystallized from hot dioxane/CCl_4; 1,2-ethanediol was stored over 4 Å molecular sieves. Salvinorin B methylthiomethyl ether **7** [2] (985 mg, 2.19 mmol) and powdered 4 Å sieves (4.1 g) were heated (60 °C) under vacuum overnight and allowed to cool under Ar. Dry CH_2Cl_2 (30 mL) and 1,2-ethanediol (3 mL) were added, and the flask cooled to 0 °C. AgOTf (741 mg, 2.6 mmol) and recrystallized NIS (630 mg, 2.6 mmol) were added in one portion and the flask purged with Ar. The resulting deep amber suspension was stirred at 0 °C for 15 min, then diluted with EtOAc (200 mL), filtered through Celite filter aid, and washed with aqueous $Na_2S_2O_3$. Brine was added to break the resulting emulsion. The organic layer was washed with sat. $NaHCO_3/H_2O$ followed by brine, then dried ($MgSO_4$), filtered and evaporated under reduced

pressure. Flash chromatography (35 µm silica gel × 8 g) in 66-100% EtOAc/hexanes gave **6** (311 mg, 0.67 mmol, 30%) as a white powder; **TLC** (EtOAc) hR_f = 38;

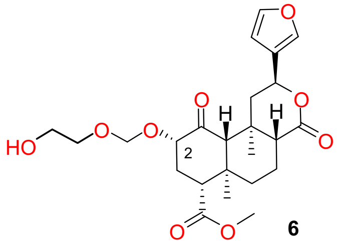

6

^{1}H NMR (300 MHz, $CDCl_3$) δ 7.42 (dt, *J* = 1.6, 0.9, 1H), 7.40 (t, *J* = 1.7, 1H), 6.38 (dd, *J* = 1.8, 0.9, 1H), 5.54 (dd, *J* = 11.7, 5.1, 1H), 4.81 (ABq, *J* = 7.3, 2H), 4.18 (dd, *J* = 11.8, 7.1, 1H), 3.80–3.68 (m, 4H), 3.71 (s, 3H), 2.69 (dd, *J* = 13.3, 3.3, 1H), 2.54 (dd, *J* = 13.3, 5.1, 1H), 2.40–2.29 (m, 2H), 2.26–2.13 (m, 2H), 2.07 (br s, 1H), 2.07-2.02 (m, 1H), 1.82–1.75 (m, 1H), 1.72–1.49 (m, 3H), 1.47 (s, 3H), 1.11 (s, 3H); **^{13}C NMR (75 MHz, $CDCl_3$)** δ 205.9, 171.7, 171.1, 143.7, 139.4, 125.3, 108.3, 95.1, 78.4, 71.9, 71.0, 64.3, 62.0, 53.8, 51.9, 51.5, 43.5, 42.0, 38.2, 35.5, 32.4, 18.1, 16.4, 15.2; **HRMS(ESI):** $[M+NH_4]^+$ *m/z* 482.2391 (calcd for $C_{24}H_{32}O_9$, 482.2384).

References

1. Wang, Y.; Tang, K.; Inan, S.; Siebert, D. J.; Holzgrabe, U.; Lee, D. Y. W.; Huang, P.; Li, J.-G.; Cowan, A.; Liu-Chen, L.-Y. *J. Pharmacol. Exp. Ther.* **2005**, *312*, 220–230. doi: 10.1124/jpet.104.073668
2. Munro, T. A.; Duncan, K. K.; Xu, W.; Wang, Y.; Liu-Chen, L.-Y.; Carlezon, W. A., Jr; Cohen, B. M.; Béguin, C. *Bioorg. Med. Chem.* **2008**, *16*, 1279-1286. doi: 10.1016/j.bmc.2007.10.067
3. Munro, T. A. The Chemistry of Salvia divinorum. Ph.D. Thesis, University of Melbourne, Australia, 2006. http://pqdtopen.proquest.com/#abstract?dispub=3304556

Table S1. Antagonism of dynorphin A (10 nM) at κ-OR in the [^{35}S]GTPγS assay.

**Antagonist (3 µM)**	**R**	[^{35}S]GTPγS binding (% increase over basal)		
				**Mean**
**None**		130	296	**213**
**JDTic**		-15	15	**0**
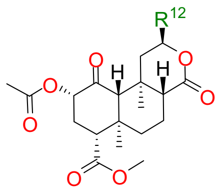	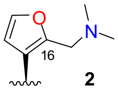 2	182	319	**251**
	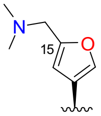 3	146	270	**208**
	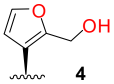 4	249	415	**332**
	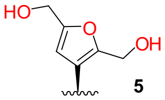 5	175	278	**226**
	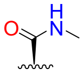	153	300	**226**
	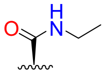	140	296	**218**
	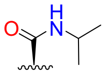	165	336	**250**
	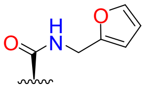	223	262	**243**
	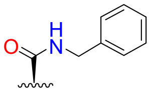	143	289	**216**
	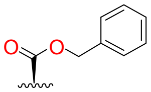	129	265	**197**
	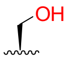	122	291	**207**
	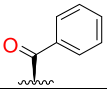	173	366	**270**
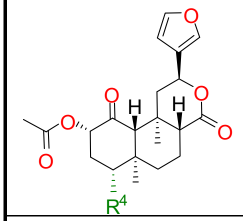	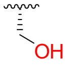	337	293	**315**
	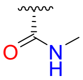	229	355	**292**
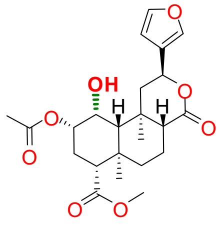		151	281	**216**

[c]Enhancement of [^{35}S]GTPγS binding to CHO-hκ-OR.

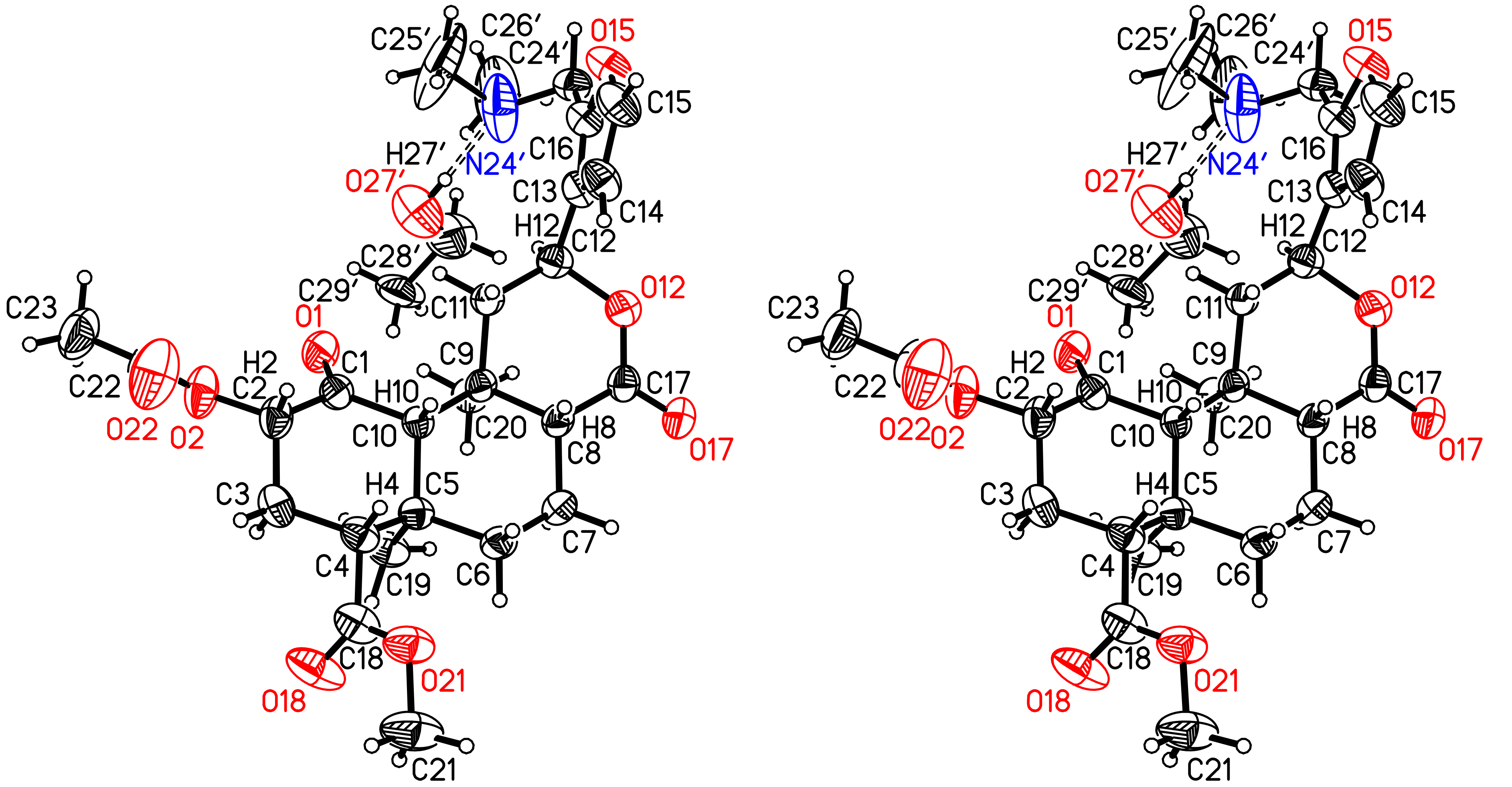

Figure S1. Crystal structure of the ethanol solvate of **2** with 50% probability thermal displacement ellipsoids (cross-eyed stereoview). The second of two orientations for the disordered dimethylamino group is shown, with an H bond to ethanol (partial occupancy).

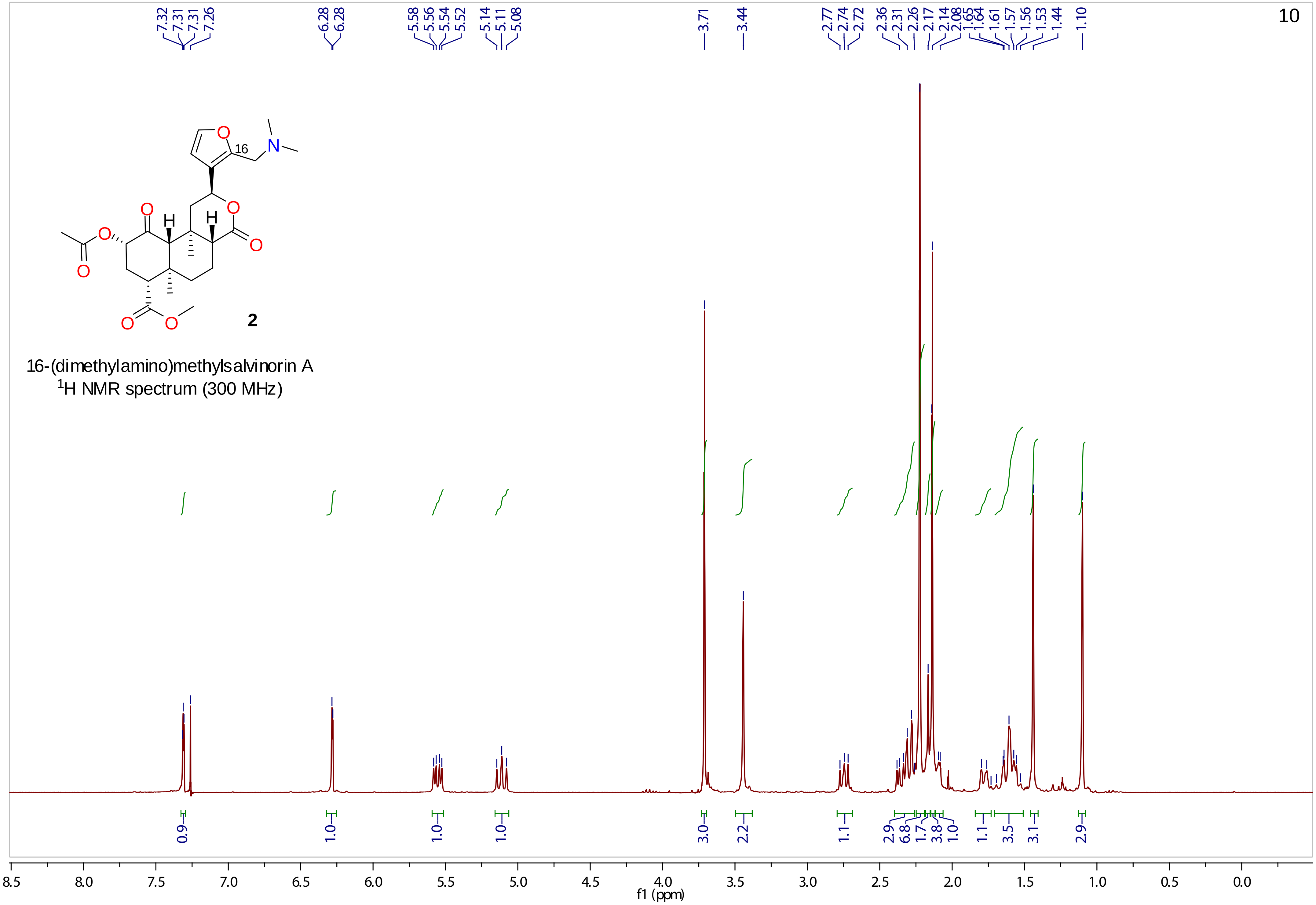
7.32
7.31
7.31
7.26
6.28
6.28
5.58
5.56
5.54
5.52
5.14
5.11
5.08
3.71
3.44
2.77
2.74
2.72
2.36
2.31
2.26
2.17
2.14
2.08
1.65
1.64
1.61
1.57
1.56
1.53
1.44
1.10
O
16
N
O
H
H
O
O
O
O
O
O
2
16-(dimethylamino)methylsalvinorin A
1H NMR spectrum (300 MHz)
0.9
1.0
1.0
1.0
3.0
2.2
1.1
2.9
6.8
1.7
3.8
1.0
1.1
3.5
3.1
2.9
8.5
8.0
7.5
7.0
6.5
6.0
5.5
5.0
4.5
4.0
3.5
3.0
2.5
2.0
1.5
1.0
0.5
0.0
f1 (ppm)

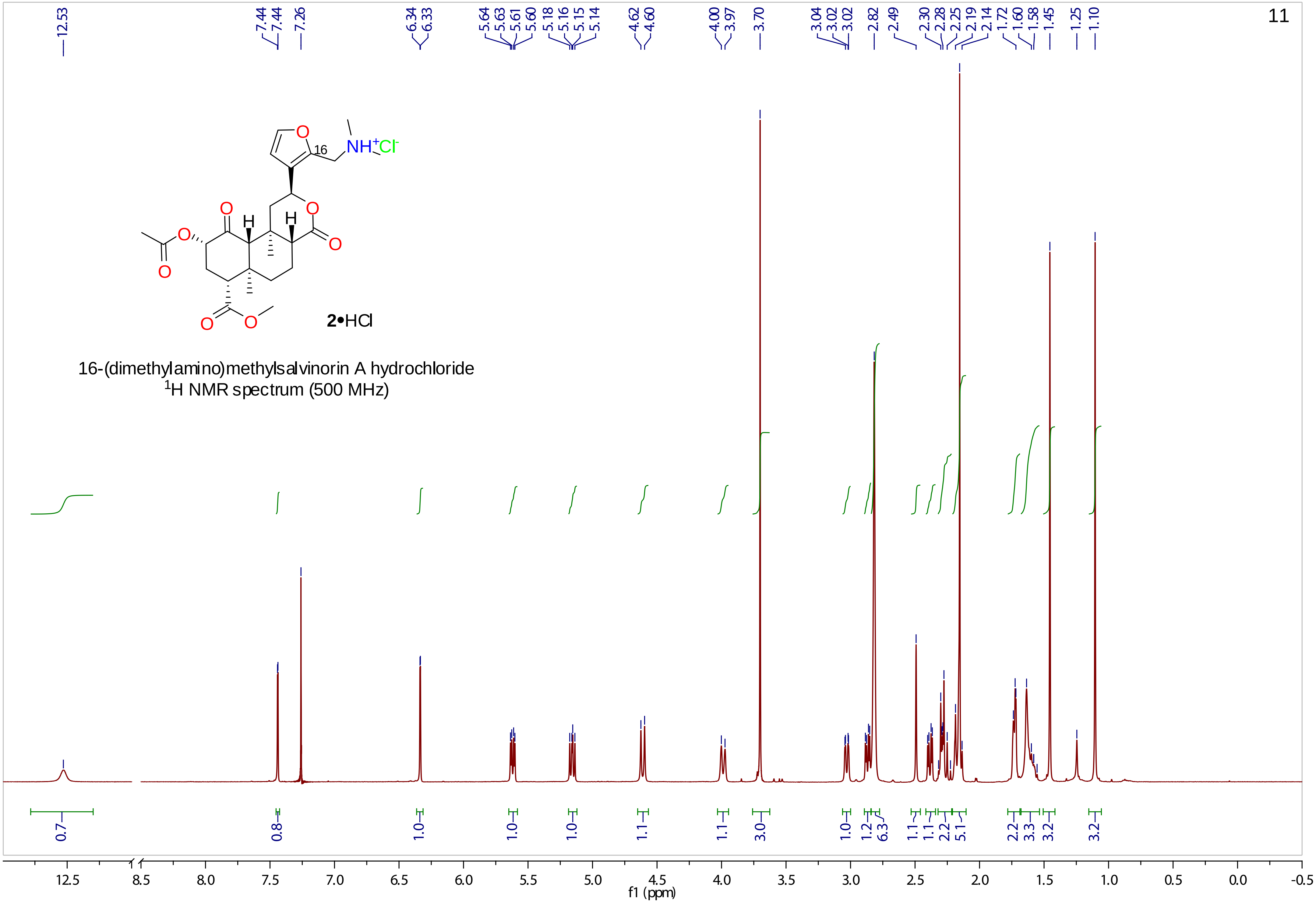
12.53
7.44
7.44
7.26
6.34
6.33
5.64
5.63
5.61
5.60
5.18
5.16
5.15
5.14
4.62
4.60
4.00
3.97
3.70
3.04
3.02
3.02
2.82
2.49
2.30
2.28
2.25
2.19
2.14
1.72
1.60
1.58
1.45
1.25
1.10
16
NH+Cl-
O
H
2•HCl
16-(dimethylamino)methylsalvinorin A hydrochloride
1H NMR spectrum (500 MHz)
0.7
0.8
1.0
1.0
1.0
1.1
1.1
3.0
1.0
1.2
6.3
1.1
1.1
2.2
5.1
2.2
3.3
3.2
3.2
f1 (ppm)

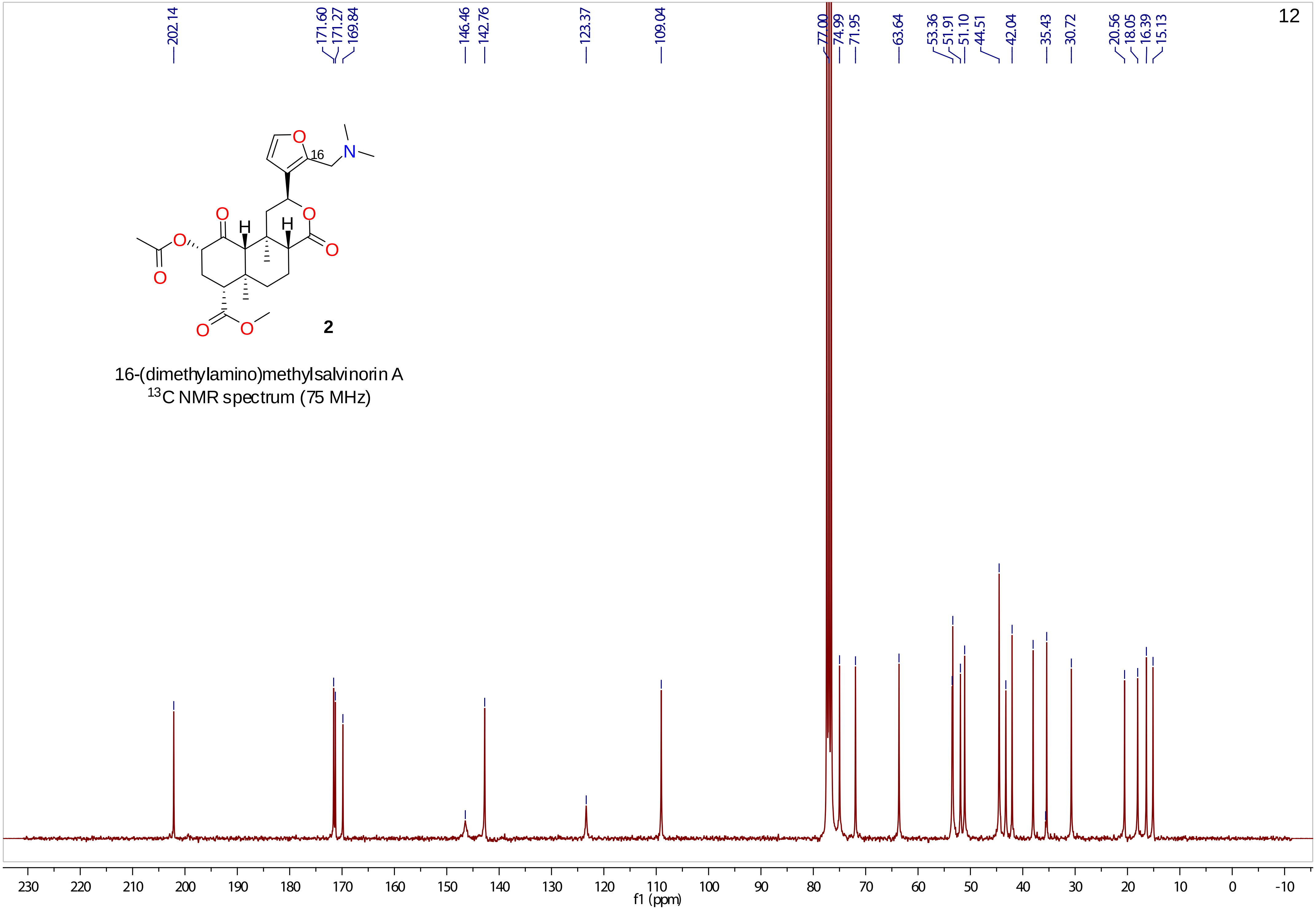
202.14
171.60
171.27
169.84
146.46
142.76
123.37
109.04
77.00
74.99
71.95
63.64
53.36
51.91
51.10
44.51
42.04
35.43
30.72
20.56
18.05
16.39
15.13
16
2
16-(dimethylamino)methylsalvinorin A
13C NMR spectrum (75 MHz)
230 220 210 200 190 180 170 160 150 140 130 120 110 100 90 80 70 60 50 40 30 20 10 0 -10
f1 (ppm)

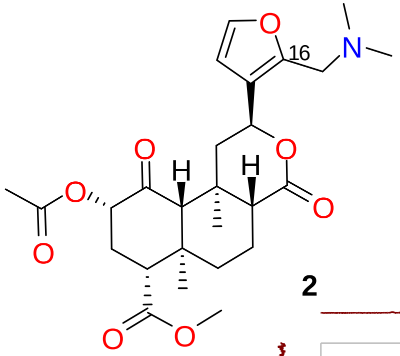

2

16-(dimethylamino)methylsalvinorin A
^{1}H-^{13}C HMQC NMR spectrum (300 MHz)

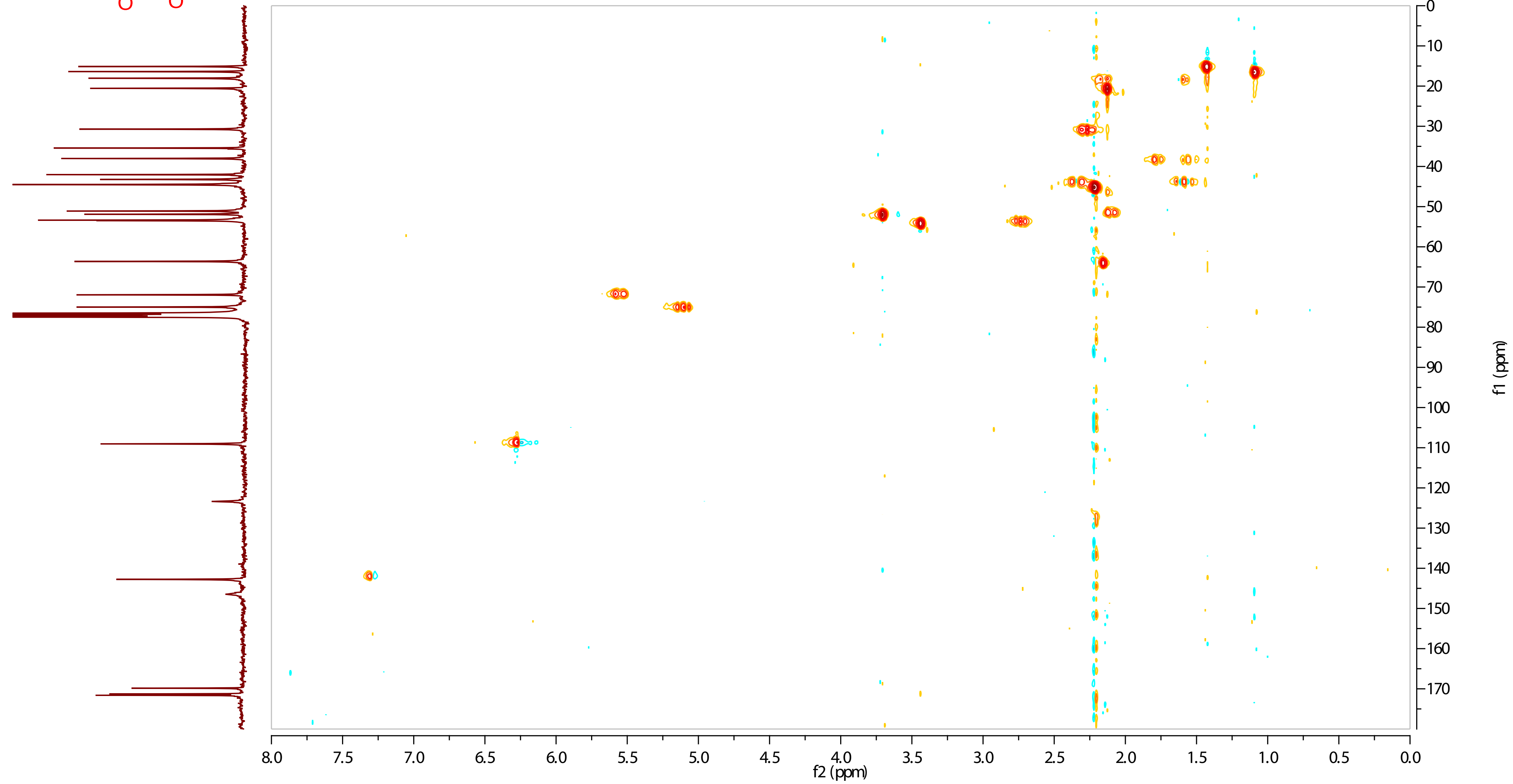

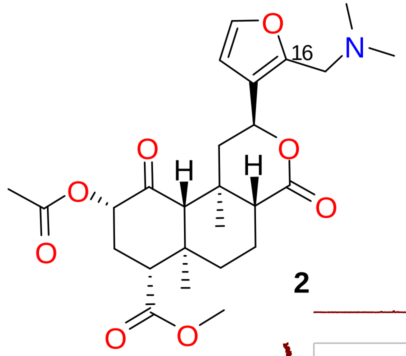

2

16-(dimethylamino)methylsalvinorin A

^{1}H-^{13}C HMBC NMR spectrum (300 MHz)

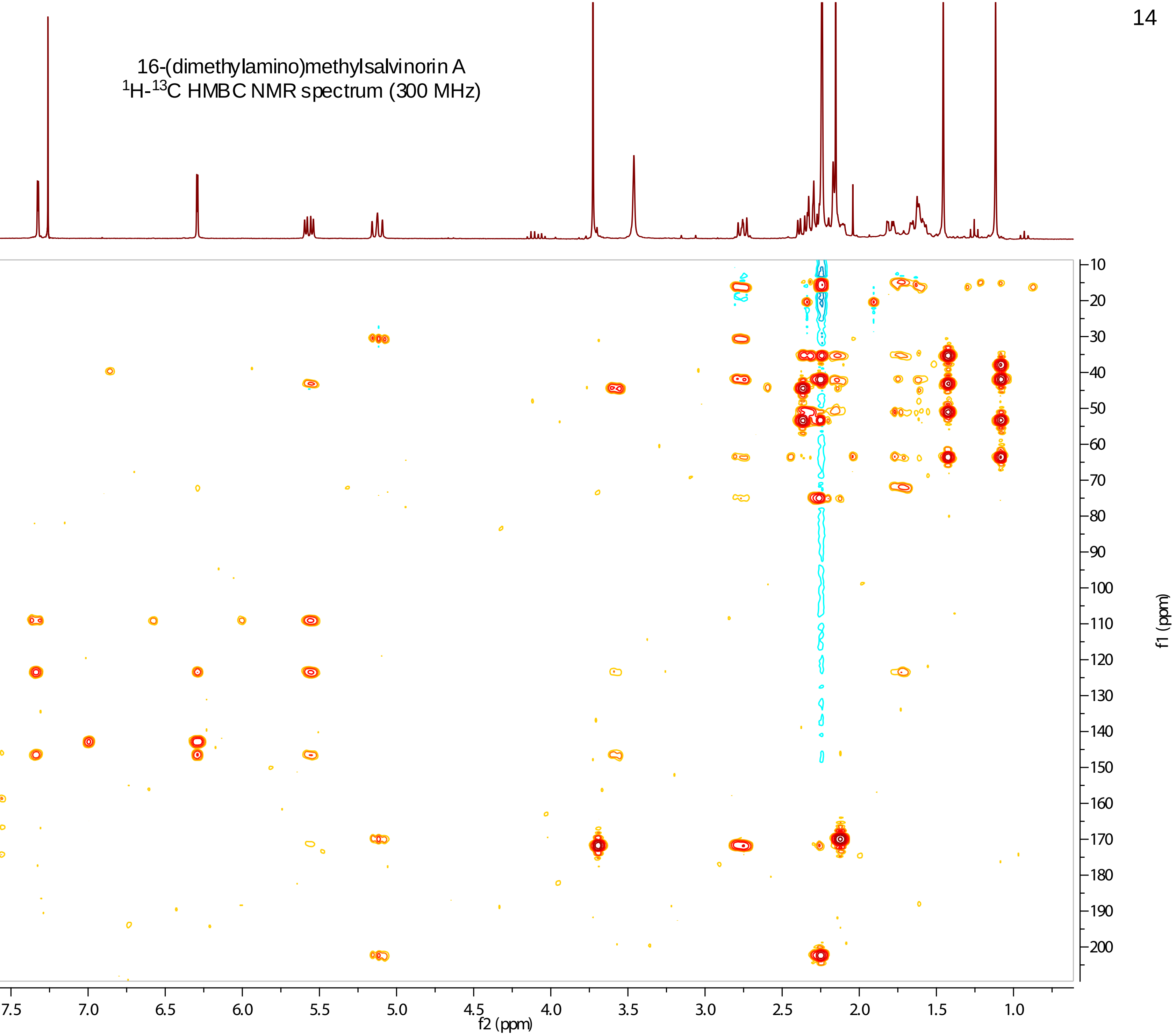

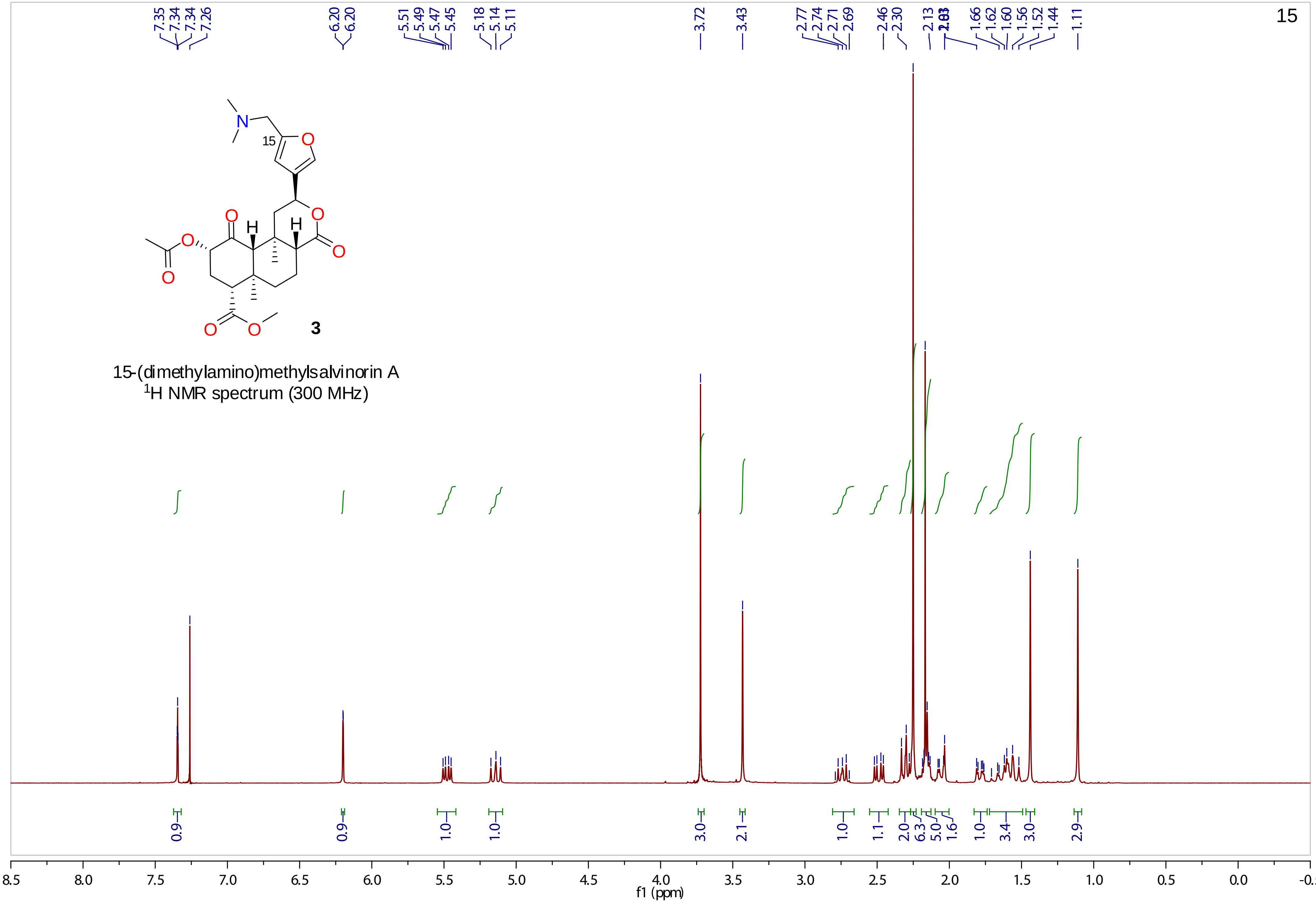

15-(dimethylamino)methylsalvinorin A
^{1}H NMR spectrum (300 MHz)

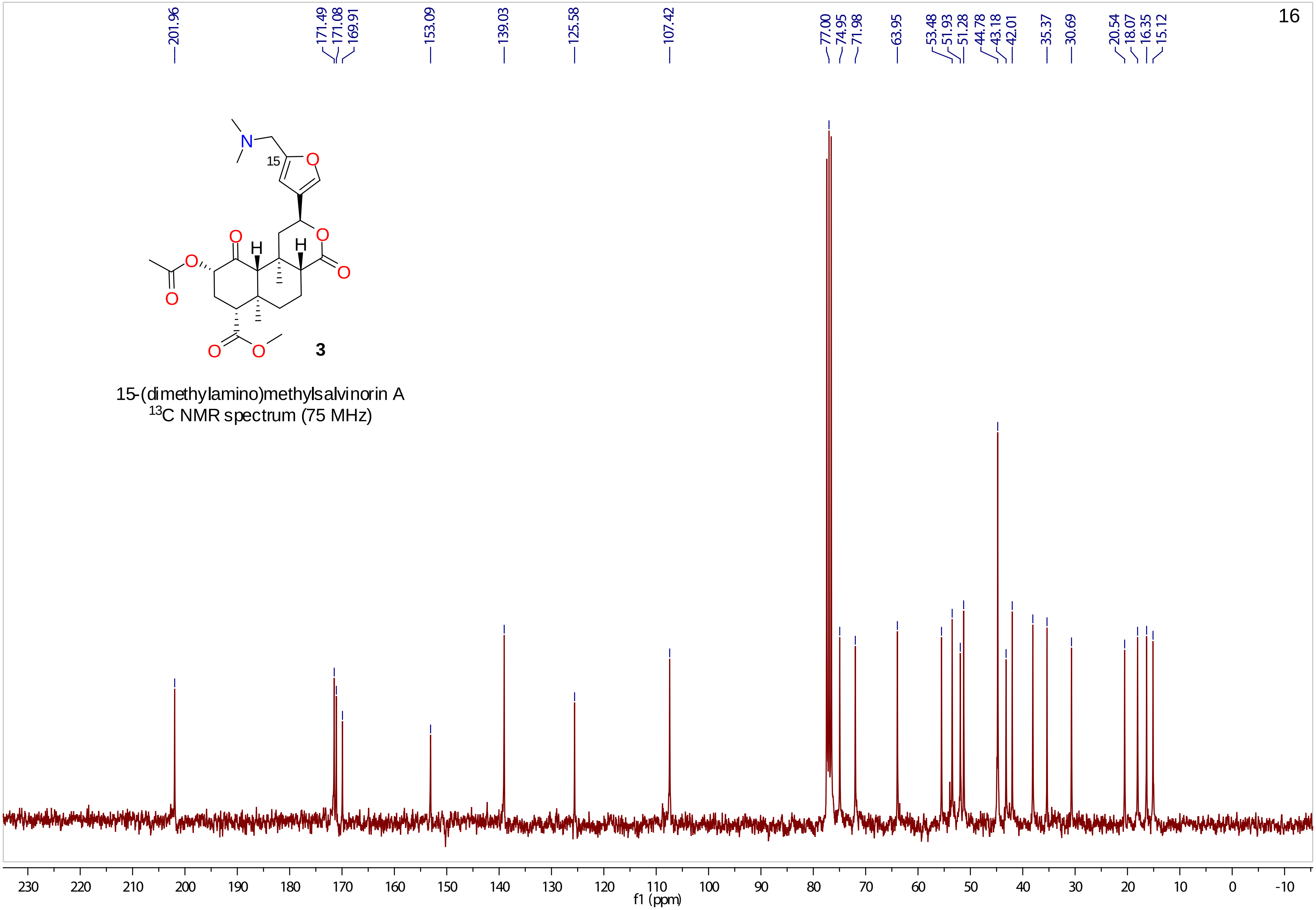
201.96
171.49
171.08
169.91
153.09
139.03
125.58
107.42
77.00
74.95
71.98
63.95
53.48
51.93
51.28
44.78
43.18
42.01
35.37
30.69
20.54
18.07
16.35
15.12
N
O
15
H
3
15-(dimethylamino)methylsalvinorin A
13C NMR spectrum (75 MHz)
230 220 210 200 190 180 170 160 150 140 130 120 110 100 90 80 70 60 50 40 30 20 10 0 -10
f1 (ppm)

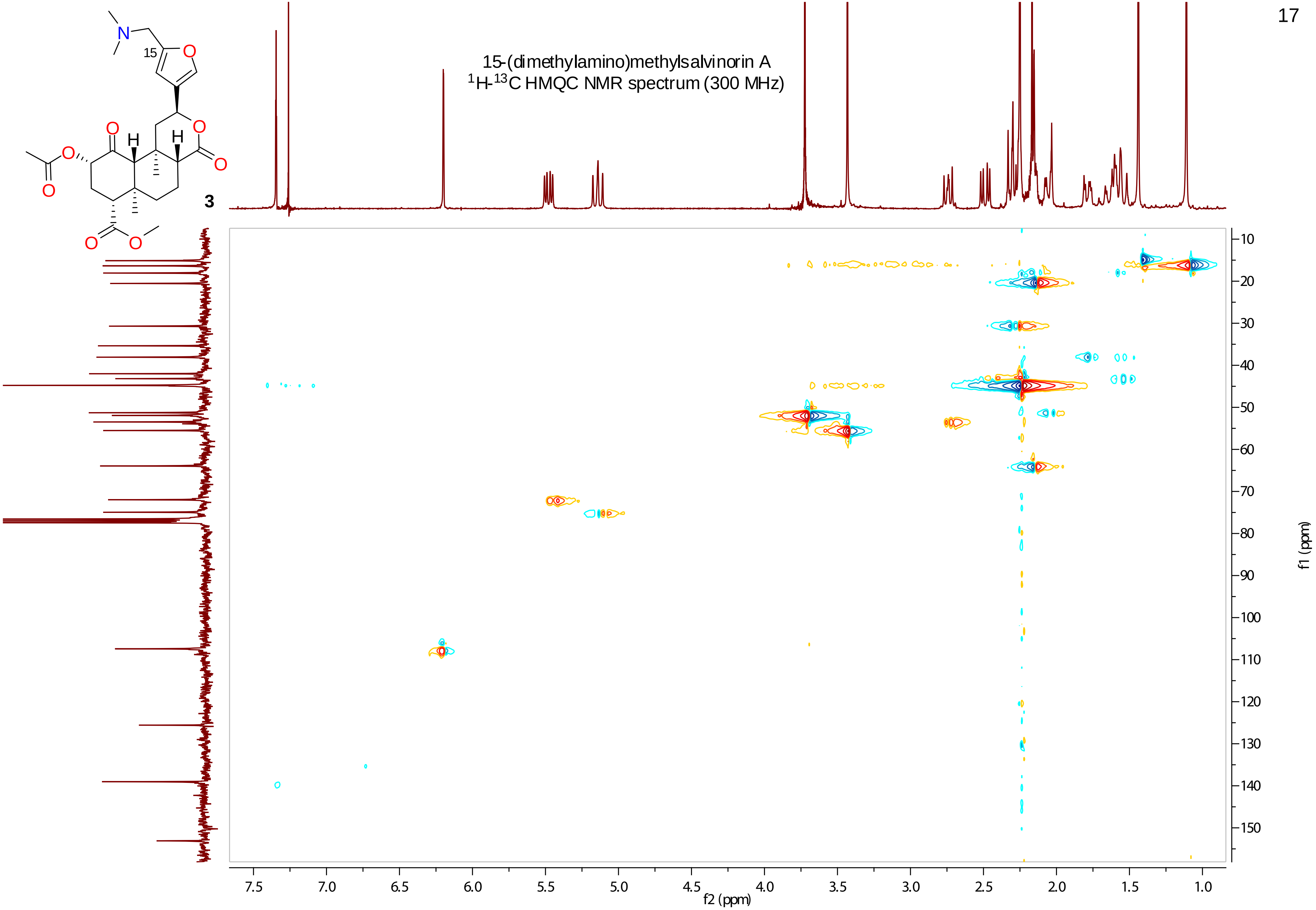
15-(dimethylamino)methylsalvinorin A
^{1}H-^{13}C HMQC NMR spectrum (300 MHz)
N
O
15
H
3
f2 (ppm)
f1 (ppm)

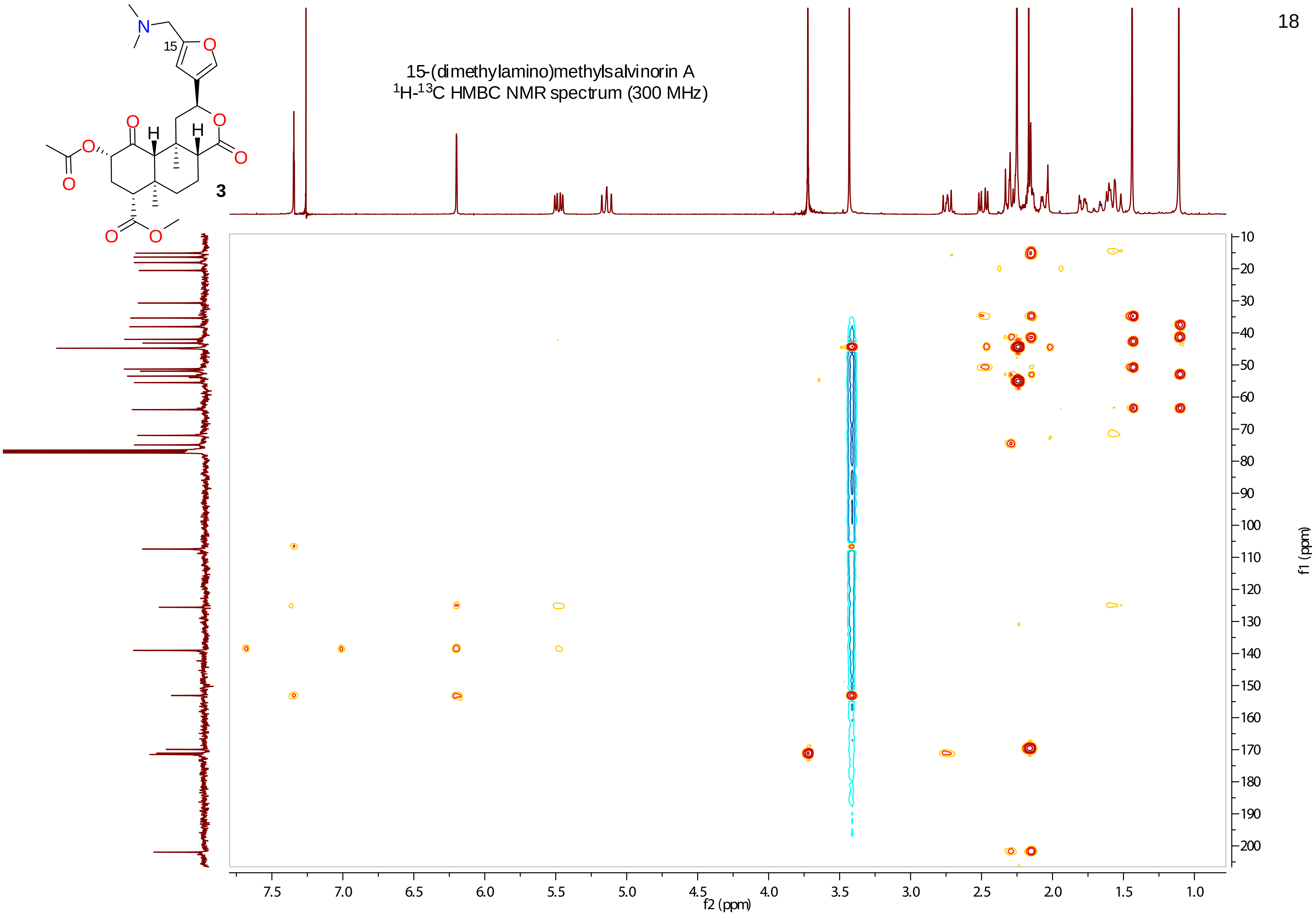
15
3
15-(dimethylamino)methylsalvinorin A
1H-13C HMBC NMR spectrum (300 MHz)
f1 (ppm)
f2 (ppm)

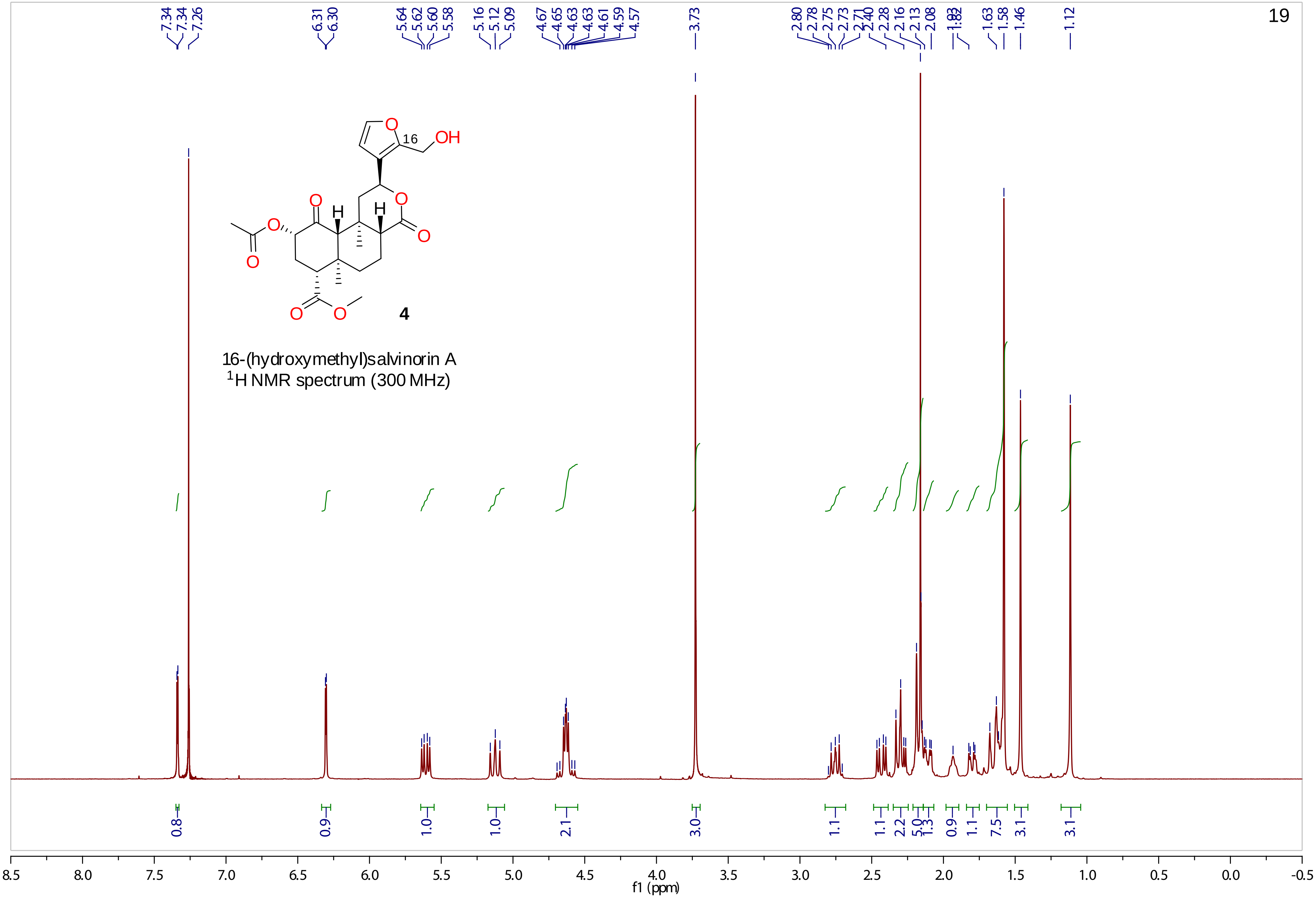

7.34
7.34
7.26
6.31
6.30
5.64
5.62
5.60
5.58
5.16
5.12
5.09
4.67
4.65
4.63
4.63
4.61
4.59
4.57
3.73
2.80
2.78
2.75
2.73
2.71
2.40
2.28
2.16
2.13
2.08
1.63
1.58
1.46
1.12
16
OH
4
16-(hydroxymethyl)salvinorin A
1H NMR spectrum (300 MHz)
0.8
0.9
1.0
1.0
2.1
3.0
1.1
1.1
2.2
0.9
1.1
7.5
3.1
3.1
f1 (ppm)

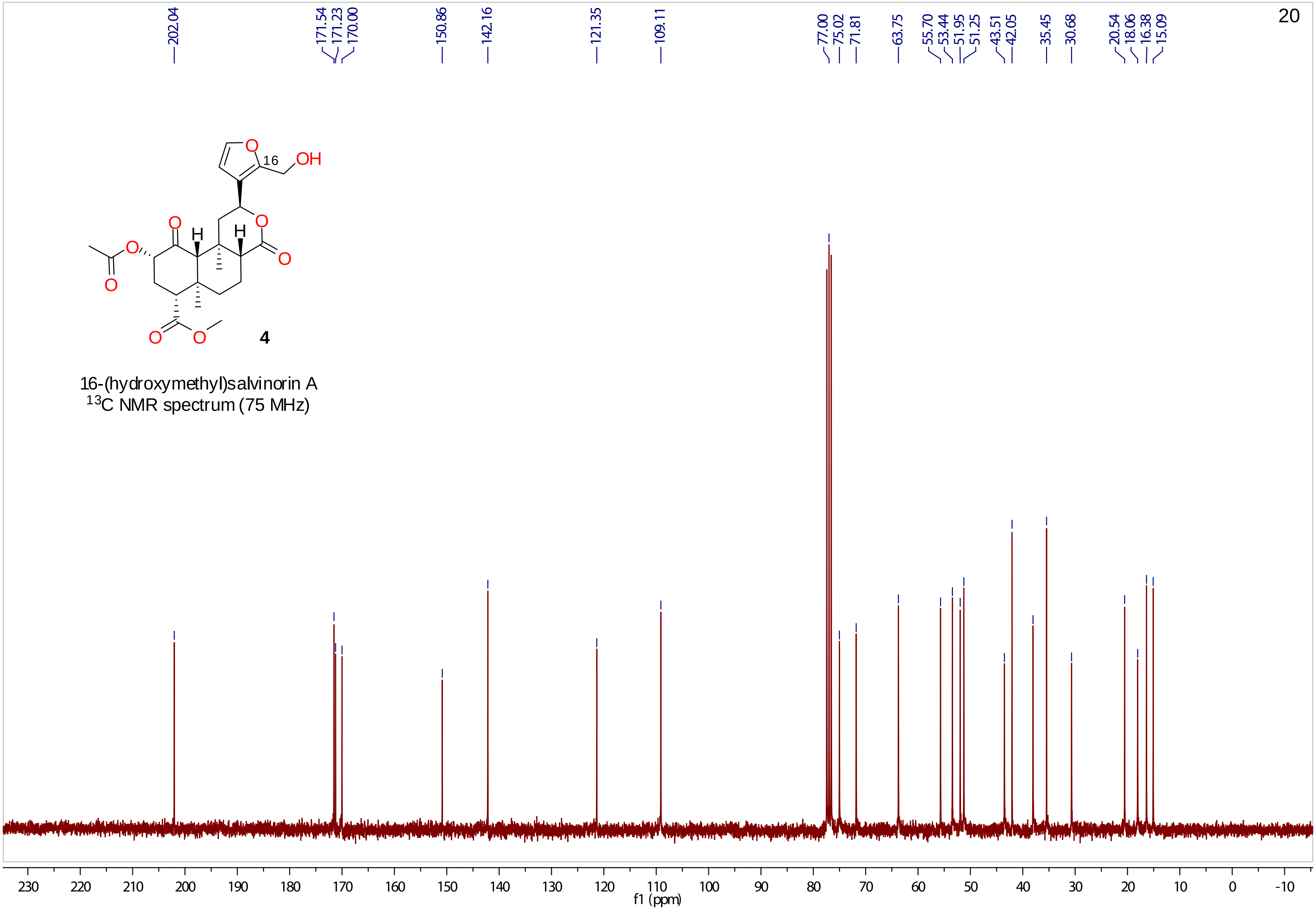
202.04
171.54
171.23
170.00
150.86
142.16
121.35
109.11
77.00
75.02
71.81
63.75
55.70
53.44
51.95
51.25
43.51
42.05
35.45
30.68
20.54
18.06
16.38
15.09
O
16
OH
O
H
H
O
O
O
O
O
O
4
16-(hydroxymethyl)salvinorin A
13C NMR spectrum (75 MHz)
230 220 210 200 190 180 170 160 150 140 130 120 110 100 90 80 70 60 50 40 30 20 10 0 -10
f1 (ppm)

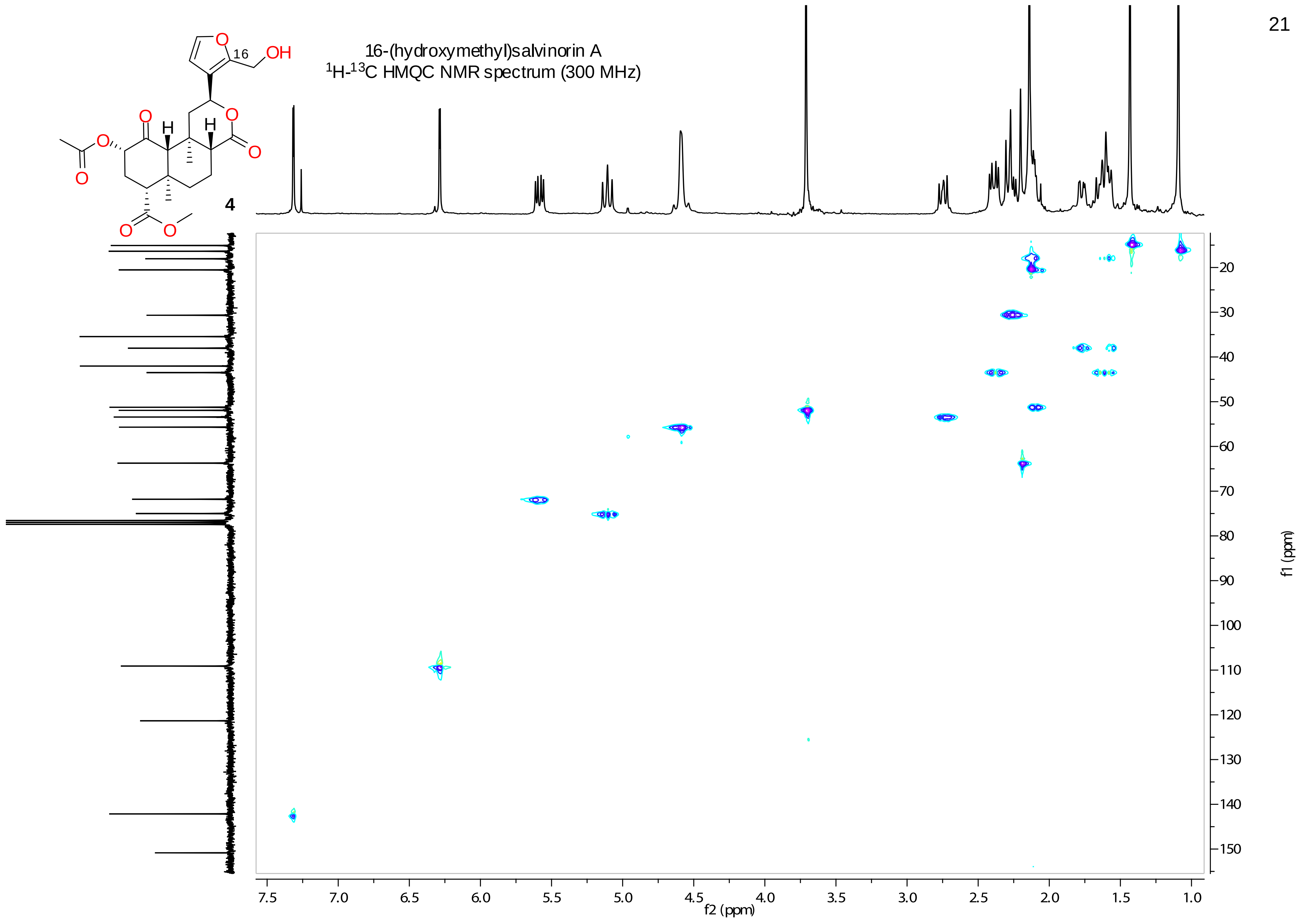
16
OH
O
H
H
4
16-(hydroxymethyl)salvinorin A
1H-13C HMQC NMR spectrum (300 MHz)
f2 (ppm)
f1 (ppm)

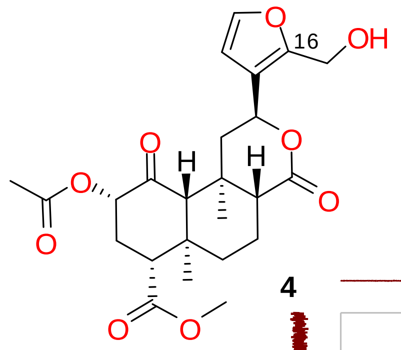

16-(hydroxymethyl)salvinorin A
^{1}H-^{13}C HMBC NMR spectrum (300 MHz)

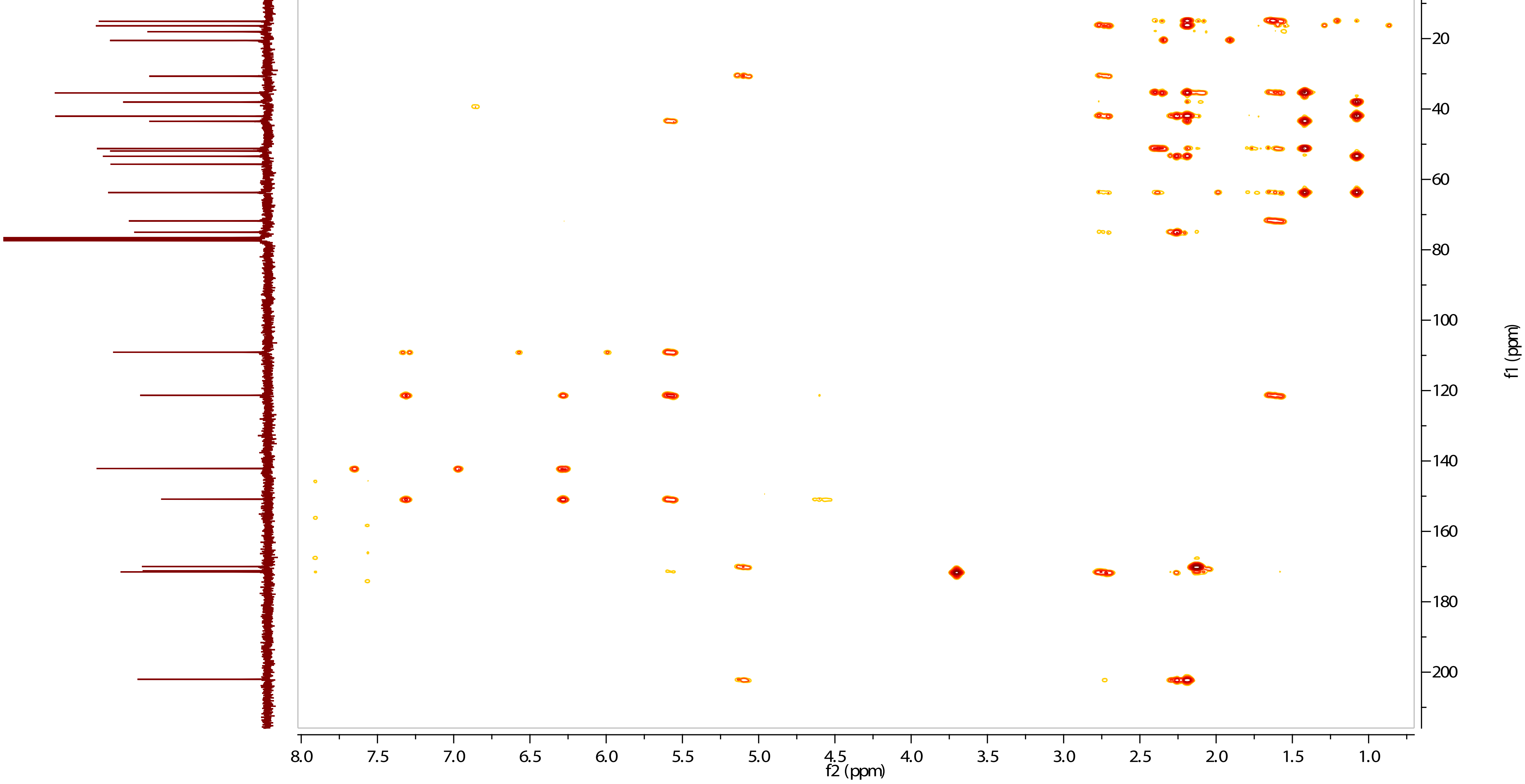

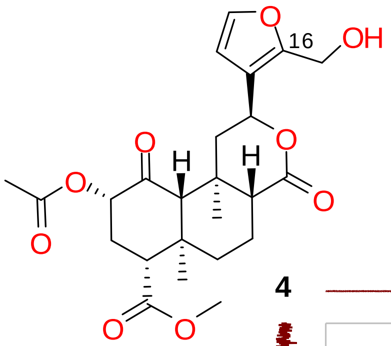

16-(hydroxymethyl)salvinorin A

^{1}H-^{13}C HMBC NMR spectrum (300 MHz) - expansion

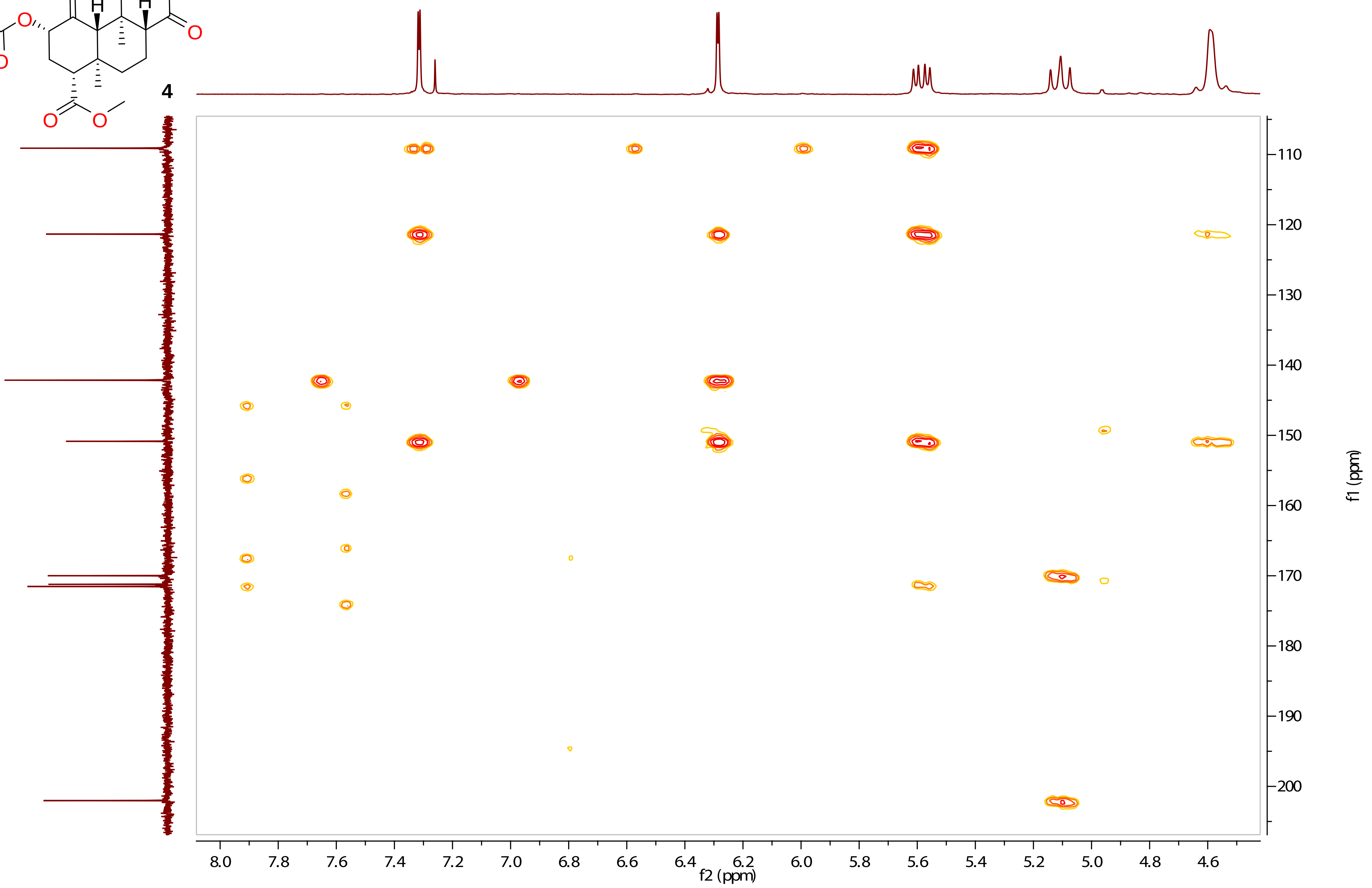

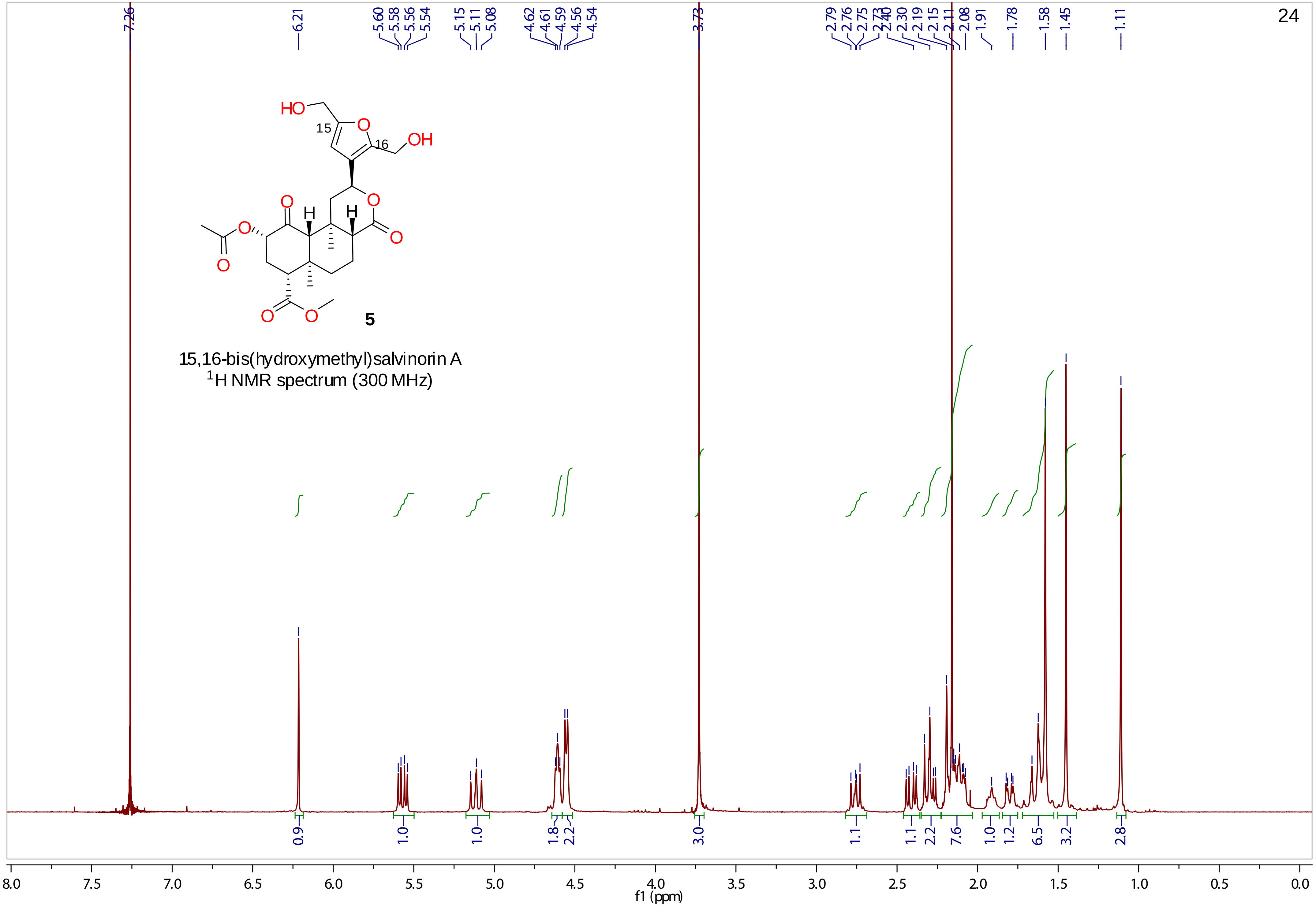
HO
OH
15
16
5
15,16-bis(hydroxymethyl)salvinorin A
1H NMR spectrum (300 MHz)
7.26
6.21
5.60
5.58
5.56
5.54
5.15
5.11
5.08
4.62
4.61
4.59
4.56
4.54
3.73
2.79
2.76
2.75
2.73
2.40
2.30
2.19
2.15
2.11
2.08
1.91
1.78
1.58
1.45
1.11
0.9
1.0
1.0
1.8
2.2
3.0
1.1
1.1
2.2
7.6
1.0
1.2
6.5
3.2
2.8
f1 (ppm)

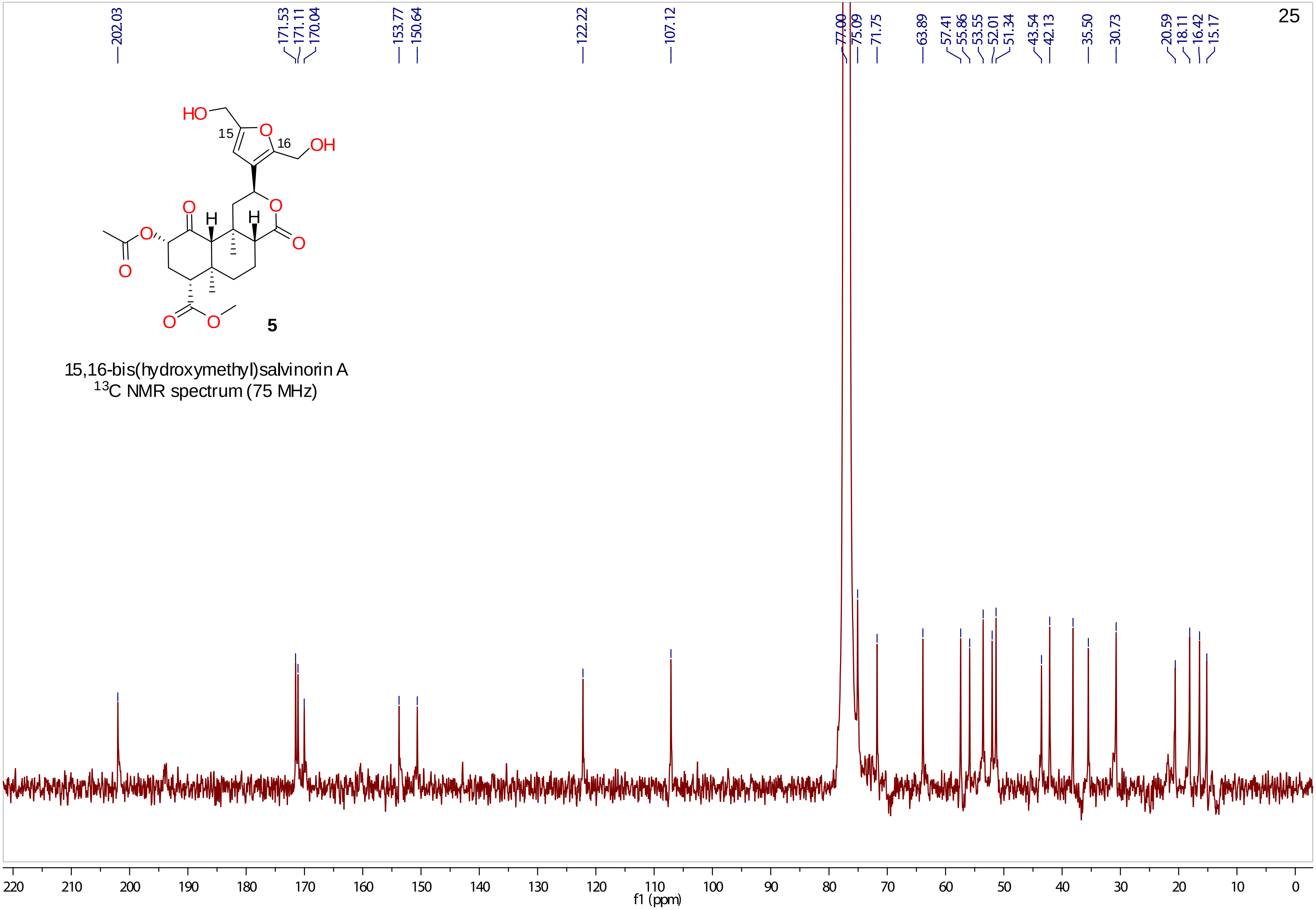
202.03
171.53
171.11
170.04
153.77
150.64
122.22
107.12
77.00
75.09
71.75
63.89
57.41
55.86
53.55
52.01
51.34
43.54
42.13
35.50
30.73
20.59
18.11
16.42
15.17
HO
15
O
16
OH
O
H
H
O
O
O
O
O
O
5
15,16-bis(hydroxymethyl)salvinorin A
13C NMR spectrum (75 MHz)
220
210
200
190
180
170
160
150
140
130
120
110
100
90
80
70
60
50
40
30
20
10
0
f1 (ppm)

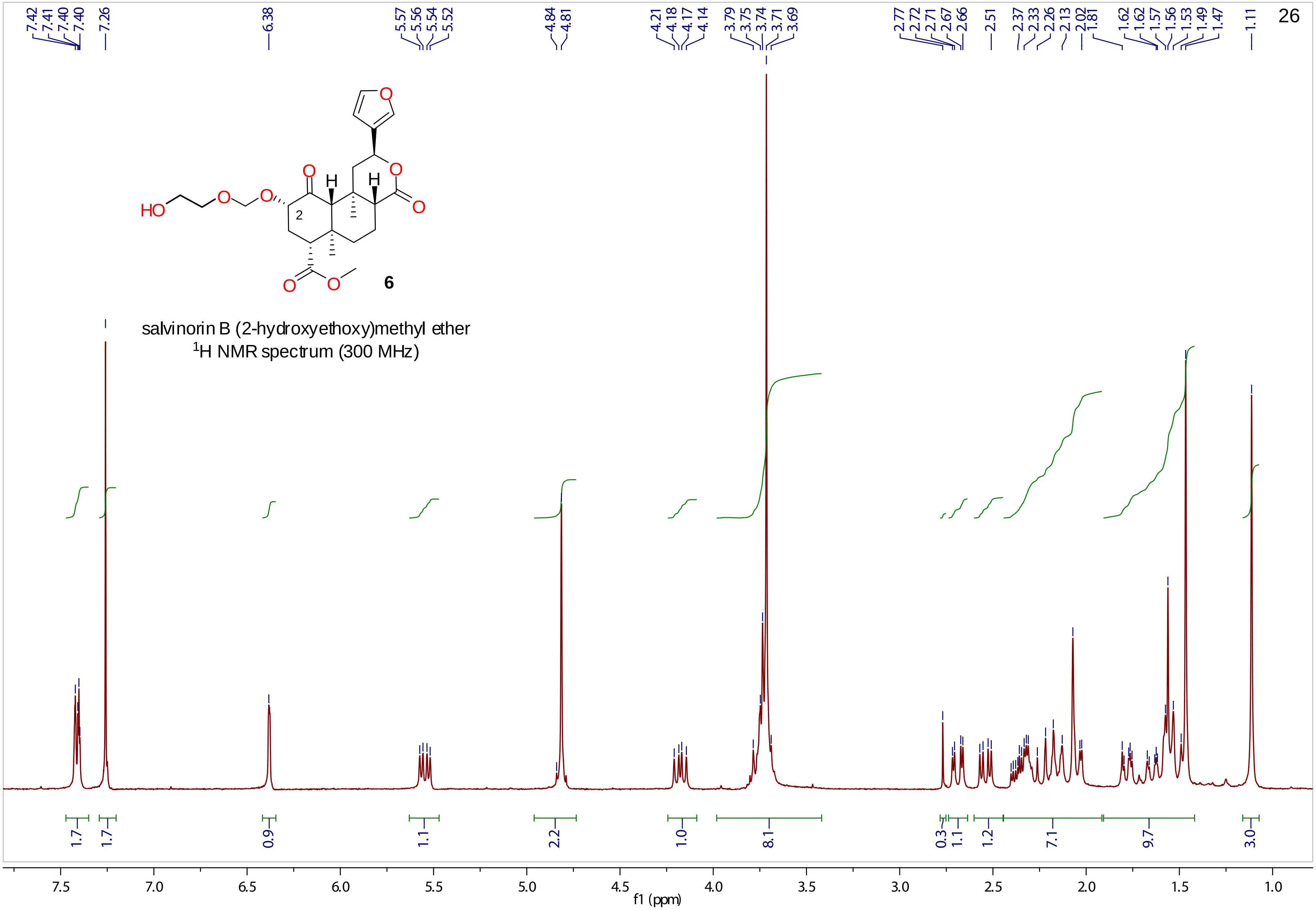
7.42 7.41 7.40 7.40 7.26 6.38 5.57 5.56 5.54 5.52 4.84 4.81 4.21 4.18 4.17 4.14 3.79 3.75 3.74 3.71 3.69 2.77 2.72 2.71 2.67 2.66 2.51 2.37 2.33 2.26 2.13 2.02 1.81 1.62 1.62 1.57 1.56 1.53 1.49 1.47 1.11
HO
O
O
O
O
O
H
H
2
O
O
6
salvinorin B (2-hydroxyethoxy)methyl ether
^{1}H NMR spectrum (300 MHz)
1.7 1.7 0.9 1.1 2.2 1.0 8.1 0.3 1.1 1.2 7.1 9.7 3.0
7.5 7.0 6.5 6.0 5.5 5.0 4.5 4.0 3.5 3.0 2.5 2.0 1.5 1.0
f1 (ppm)

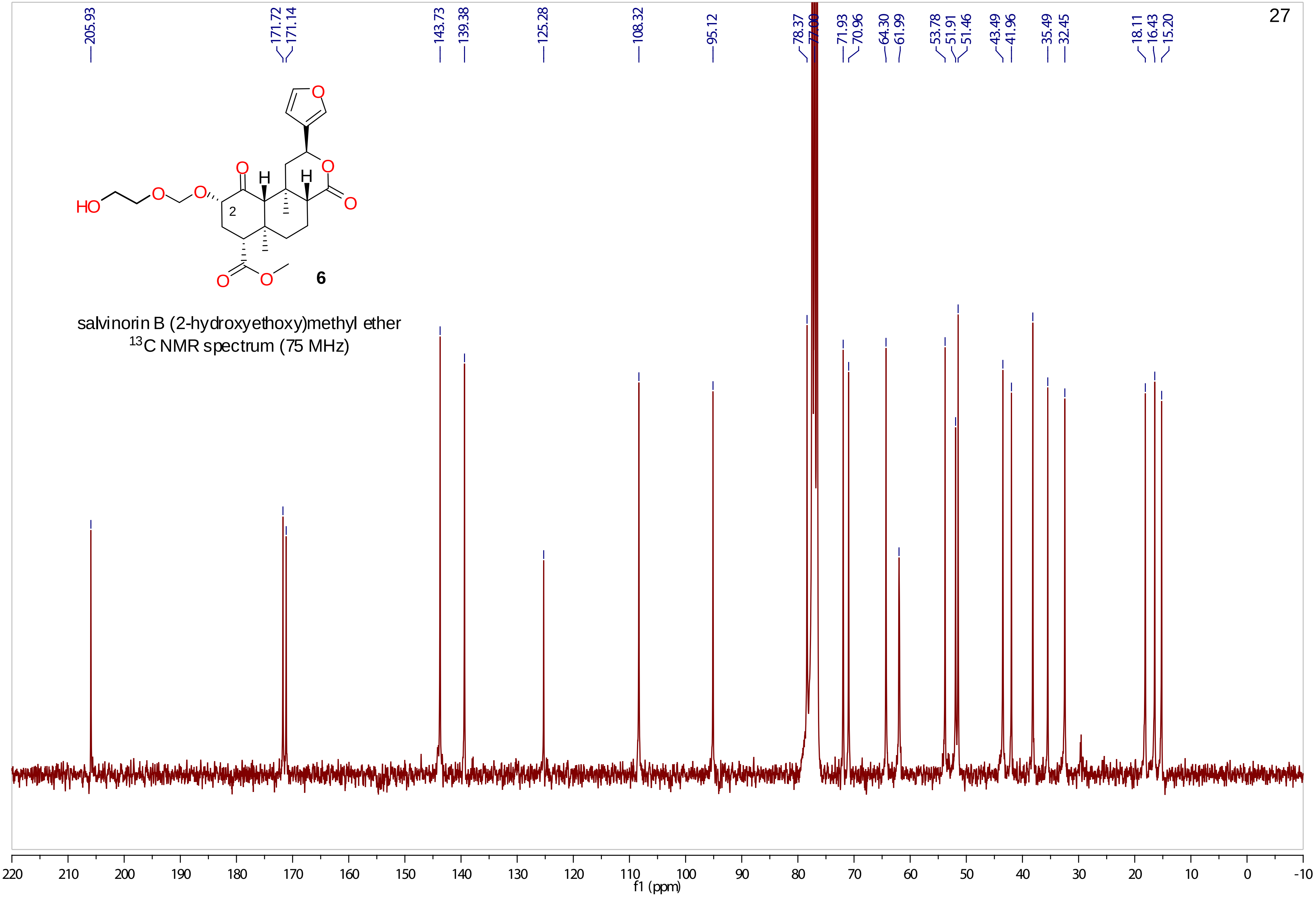
205.93
171.72
171.14
143.73
139.38
125.28
108.32
95.12
78.37
77.00
71.93
70.96
64.30
61.99
53.78
51.91
51.46
43.49
41.96
35.49
32.45
18.11
16.43
15.20
HO
O
O
O
H
H
O
O
O
2
O
O
6
salvinorin B (2-hydroxyethoxy)methyl ether
13C NMR spectrum (75 MHz)
f1 (ppm)
